# Bipolar membrane electrolyzers enable high single-pass CO_2_ electroreduction to multicarbon products

**DOI:** 10.1038/s41467-022-31295-3

**Published:** 2022-06-24

**Authors:** Ke Xie, Rui Kai Miao, Adnan Ozden, Shijie Liu, Zhu Chen, Cao-Thang Dinh, Jianan Erick Huang, Qiucheng Xu, Christine M. Gabardo, Geonhui Lee, Jonathan P. Edwards, Colin P. O’Brien, Shannon W. Boettcher, David Sinton, Edward H. Sargent

**Affiliations:** 1 Department of Electrical and Computer Engineering, University of Toronto, 10 King’s College Road, Toronto, ON M5S 3G4 Canada; 2 Department of Mechanical and Industrial Engineering, University of Toronto, 5 King’s College Road, Toronto, ON M5S 3G8 Canada; 3 Department of Chemical Engineering, Queen’s University, 19 Division Street, Kingston, Kingston, ON K7L 3N6 Canada; 4 Department of Chemistry and Biochemistry, University of Oregon, Eugene, OR 97403 USA

**Keywords:** Electrocatalysis, Carbon capture and storage

## Abstract

In alkaline and neutral MEA CO_2_ electrolyzers, CO_2_ rapidly converts to (bi)carbonate, imposing a significant energy penalty arising from separating CO_2_ from the anode gas outlets. Here we report a CO_2_ electrolyzer uses a bipolar membrane (BPM) to convert (bi)carbonate back to CO_2_, preventing crossover; and that surpasses the single-pass utilization (SPU) limit (25% for multi-carbon products, C_2+_) suffered by previous neutral-media electrolyzers. We employ a stationary unbuffered catholyte layer between BPM and cathode to promote C_2+_ products while ensuring that (bi)carbonate is converted back, in situ, to CO_2_ near the cathode. We develop a model that enables the design of the catholyte layer, finding that limiting the diffusion path length of reverted CO_2_ to ~10 μm balances the CO_2_ diffusion flux with the regeneration rate. We report a single-pass CO_2_ utilization of 78%, which lowers the energy associated with downstream separation of CO_2_ by 10× compared with past systems.

## Abstract (web summary)

In the carbon dioxide (CO_2_) to multicarbon electrolysis, the crossover CO_2_ to the oxygen-rich anodic gas stream add a further energy-intensive chemical separation step. Here, the authors demonstrate a bipolar membrane-based electrolyzer design that eliminates the crossover CO2.

## Introduction

CO_2_RR for C_2+_ production requires the simultaneous achievement of high production rate and high energy efficiency^1,2^. The current densities in flow cells (A in Table 1) and membrane-electrode assemblies (MEAs, B in Table 1) have reached industrially relevant levels (ethylene partial current density > 100 mA cm^−2^)^3^; however, the energy penalty associated with low single-pass CO_2_ utilization (SPU: the fraction of the CO_2_ converted to the total input CO_2_) has yet to be reduced to practical levels (SPU > 40%)^4^. Carbonate formation and crossover in typical CO_2_RR electrolyzers limit the SPU to ≤25% for C_2+_ (details in SI1), imposing energy penalties of 280–480 GJ in alkaline-media, and of 80–130 GJ in neutral-media, for the production of each ton of ethylene^5,6^.

**Table 1:** Comparison of the different electrolyzer designs employed in CO_2_RR.

	Configuration diagrams	Cathode products	CO_2_-originated carbonate mass balance	Catholyte	Cathode micro-environment	Max. total SPU for Cu cathode	Ref.
A		C_1_ and C_2+_	Loss in catholyte; migrate through AEM and reverted to CO_2_ at anode	OH^−^, HCO_3_^−^, SO_4_^2−^	Locally strong alkaline, with bulk alkaline or neutral catholyte	24%	^21,41^
B		C_1_ and C_2+_	Migrate through AEM and revert to CO_2_ at anode	Solid-state polymer (AEM)	Locally strong alkaline	30%	^21^
C		C_1_	Revert to CO_2_ at the surface of CEL	Solid-state polymer (PAA-PAH bilayers)	Locally weak acidic	N/A	^42^
D		C_1_	Revert to CO_2_ at the surface of CEL	Solid-state polymer (CEL of BPM)	Locally strong acidic, cation effect	N/A	^10^
E		C_1_ and C_2+_ (C_2+_ shown in SI3)	Revert to CO_2_ at the surface of CEL	>600 μm thick NaHCO_3_	Locally strong alkaline, with bulk neutral catholyte	15%	^12^
F		C_1_ and C_2+_	Reverted to CO_2_ near (~12 μm) the cathode	65 μm thick K_2_SO_4_	Locally strong alkaline, with bulk neutral catholyte	78%	This work

Analysis of the CO_2_ and carbonate mass balance in neutral-media electrolyzers (SI1) indicates that achieving high SPU requires that (bi)carbonate not cross the membrane to the anode, and that (bi)carbonate formed at the cathode must revert to CO_2_ and remain available to participate in CO_2_RR by returning to the cathode.

Bipolar membranes (BPMs) have been used to block CO_2_ crossover and convert (bi)carbonate back to CO_2_^7–9^. In CO_2_RR electrolyzers, commercial BPMs inhibit CO_2_ loss^10^, but the acidic cation-exchange layer (CEL) degrades the cathode’s CO_2_RR selectivity^10^.

**Fig. 1:**
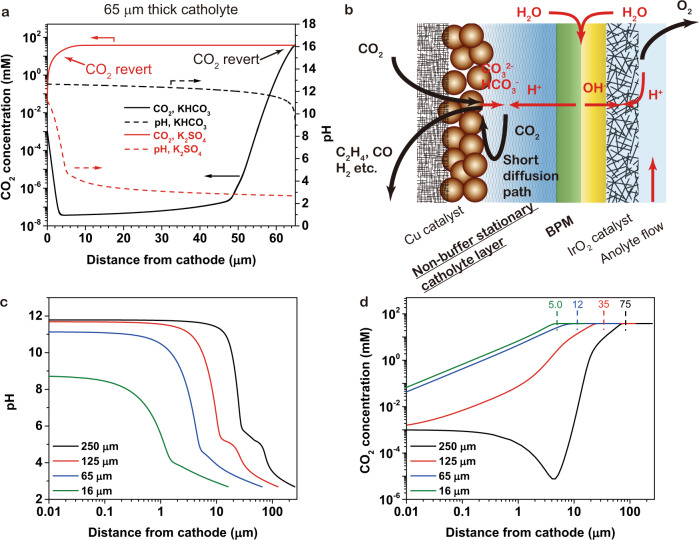
The configuration and simulation of the electrolyzer design. **a** The CO_2_ (solid lines) and pH distributions (dashed lines) in the 65 μm-thick SC-layer. The positions where the (bi)carbonates revert to CO_2_ are marked (red for non-buffering and black for buffering electrolyte). **b** The schemes and the mass transfer in the SC-BPMEA. **c** The pH distribution inside the SC-layer. **d** The dissolved CO_2_ concentration profile inside the SC-layer. The virtual boundaries marked by dash lines are defined as the position where the CO_2_ concentration becomes 1% lower than the bulk concentration. The numbers marked above are the distances between the virtual boundaries and the cathode surface.

To address cathode acidification, one may use a buffering catholyte (e.g., KHCO_3_) between CEL and cathode^9,11,12^; this approach (Fig. 1a) provides a pH at the CEL surface of ~3, and keeps the cathodic local pH >12. However, in a buffering catholyte, (bi)carbonate reverts to CO_2_ near the surface of the CEL (solid black line in Fig. 1a), slowing mass transfer of the reverted CO_2_ and reducing reactant availability for CO_2_RR. This leads to the best SPUs reported of ~15% in prior BPM-based electrolyzers in C_2+_ electroproduction (SI3 and SI5). When the catholyte is flowing, the SPU is even lower, ~6% according to previous reports, because flowing catholyte removes reverted CO_2_^11^. Prior BPM-based electrolyzers (Table 1) have, as a result, not exceeded the 25% SPU limitation for C_2+_ electroproduction (SI3).

We show that a stationary, non-buffering catholyte with well-designed catholyte layer thickness (Fig. 1b) addresses these mass-transfer limitations while simultaneously providing the needed high local cathode pH (Fig. 1a). BPM-generated protons migrate farther in non-buffering catholyte than in buffering catholyte, since the latter consumes protons at a higher rate. Here we show further quantitative analysis of the design requirements to achieve this target.

## Results

### Finite-element numerical simulations of the stationary catholyte (SC)-layer

The composition and thickness of the catholyte layer influence the local pH, the efficiency of CO_2_ regeneration and, thereby, the overall cell performance. We applied a one-dimensional multiphysics model in COMSOL to investigate the catholyte layer in BPM-based CO_2_RR electrolyzers.

The CO_2_ reactant is provided by two sources: the inlet CO_2_ flow (gas) and the regenerated CO_2_ (dissolved form, aq.) in the catholyte. To achieve high SPU, it is necessary to restrict the gaseous CO_2_ feed^13,14^. Under a restricted gaseous CO_2_ availability, the cathode CO_2_ supply relies more on regeneration (SI1): in an ideal case with 100% SPU and 100% C_2+_ selectivity, regeneration contributes 75% of the consumed CO_2_. Thus, the mass transport of regenerated CO_2_ is most critical, and that transport is governed by catholyte composition and thickness.

At steady-state, electrolysis creates a pH gradient through the catholyte layer: the pH is high near the cathode and low near the CEL. The protons and (bi)carbonate ions recombine in the catholyte, forming CO_2_ (aq.) that diffuses, in response to a concentration gradient, to the Cu catalyst.

Simulations resolve the local cathode environment as a function of dimensions, electrolyte, and running conditions (Figs. 1a, c, d, SI4 and SI5). We selected 250, 125, 65, and 16 μm as the modelled thicknesses to correspond to commercially available materials.

Use of a buffering catholyte (e.g., KHCO_3_) leads to a thick CO_2_ (aq.) diffusion layer close to the catholyte thickness, since the CO_2_ (aq.) is generated near the CEL surface, as shown in Fig. 1a (details in SI5). This effect reduces the CO_2_ (aq.) mass-transfer efficiency. The experimental results presented and discussed in SI6 show similar trends to the simulation results.

In contrast, Fig. 1a, c illustrate that with a non-buffering catholyte layer (e.g., 0.5 M K_2_SO_4_) with thicknesses of 250, 125, and 65 μm, the local pH values near the cathode are greater than 11, which is sufficient to promote selectivity towards CO_2_RR over HER^13^. Reducing the SC-layer thickness to 16 μm results in a cathode pH of 8.7, implying a lower selectivity toward CO_2_RR.

Figure 1c, d shows the simulated concentration profiles of CO_2_ (aq.) in the non-buffering SC-layer. At steady-state, the CO_2_ (aq.) is continuously supplied to the cathode to participate in CO_2_RR, forming a concentration gradient (the boundary was defined here as the position where CO_2_ concentration is 1% lower than the saturated concentration) to the cathode surface. Prior studies have termed the zone between the cathode and this boundary the diffusion layer^15^. The thickness of the diffusion layer controls the efficiency of CO_2_ (aq.) mass transport^15^. According to the simulations, the thicknesses of the diffusion layers are 75, 35, 12, and 5 μm for the catholyte layers with the thicknesses of 250, 125, 65, and 16 μm, respectively (marked in Fig. 1d). For reference, the CO_2_ (aq.) diffusion layer thickness in H-cells (all CO_2_ supplied in dissolved form) is typically 40–100 μm, and this does not support current densities exceeding 100 mA cm^−2^.^16^ We expect that diffusion layers <40 μm, and a corresponding catholyte thickness <150 μm, are required for sufficient mass transport in a non-buffering catholyte. To achieve similar mass transport in a buffering catholyte, the total thickness could not exceed 12 μm, and the cathodic pH would not be sufficiently alkaline for selective CO_2_RR (SI5).

The simulation results suggest the following design principles for the catholyte layer in a BPM-based electrolyzer: the local cathode pH and the diffusion layer thickness of the regenerated CO_2_ increase as the catholyte thickness increases; the buffering capacity of the catholyte increases the diffusion layer thickness and reduces transport. Precise control of the thickness of a non-buffering catholyte should thus offer a route to high SPU, CO_2_RR selectivity, and reaction rate.

### System design for high SPU of CO_2_ feedstock

Guided by the above analysis, we focused on a *stationary catholyte bipolar membrane electrode assembly* (SC-BPMEA) electrolyzer and incorporated a judiciously-designed catholyte layer and BPM (Fig. 1a).

**Fig. 2:**
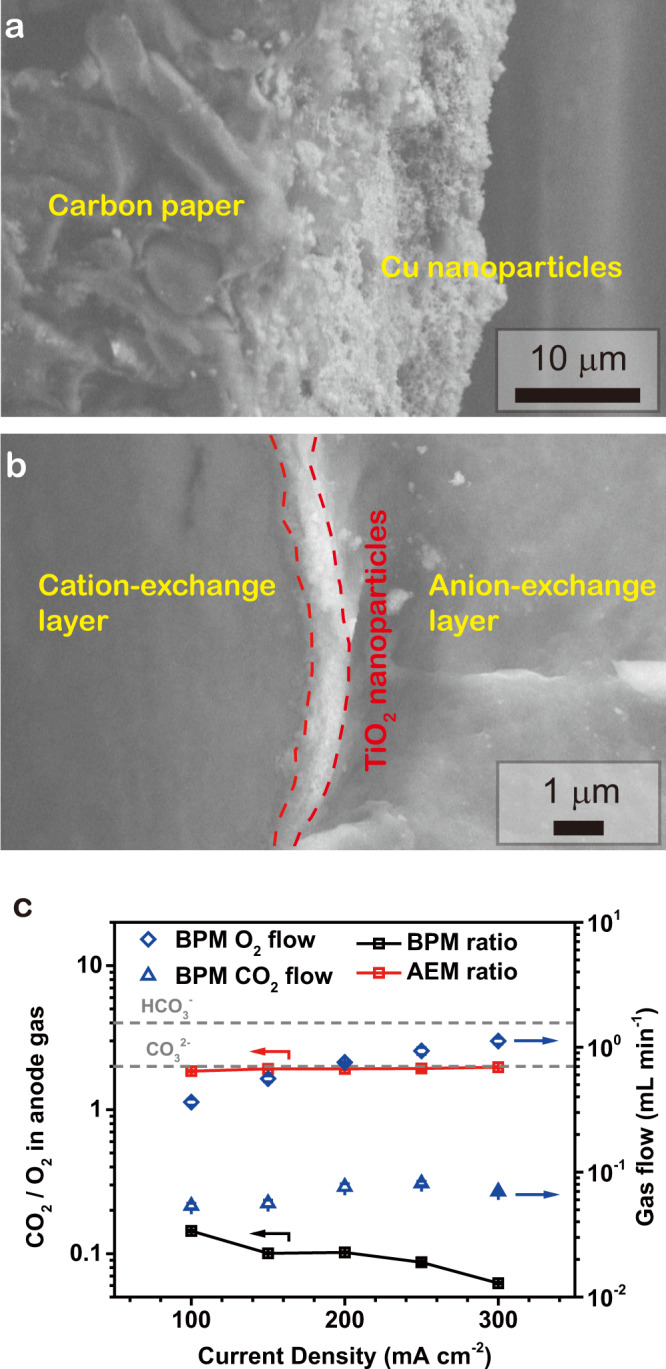
Characterization of electrodes and BPM. Scanning electron microscopy (SEM) images of the cathode electrode (**a**) and the CEL/AEL interface of the custom BPM used for SC-BPMEA in neutral 0.1 M KHCO_3_ anolyte (**b**). The custom BPM consists of a Nafion^TM^ 212 as CEL, Piperion (Versogen) as AEL, and a TiO_2_ nanoparticle layer sandwiched in between as the water dissociation catalyst. **c** The CO_2_/O_2_ ratio in the anode gas stream for the conventional electrolyzers (red squares) and our SC-BPMEA (black squares) at various current densities. O_2_ and CO_2_ flow rates in the SC-BPMEA are also indicated. The plots show data obtained after 1 h of continuous electrolysis at each current density. The data are from an SC-BPMEA with a 125 μm-thick SC-layer. Similar data were collected for 65 and 250 μm layers (difference below 5%). The error bars represent the standard deviation of three measurements.

The cathode was prepared by spraying Cu nanoparticles onto a hydrophobic carbon gas-diffusion layer for CO_2_RR (Fig. 2a). The anode was IrO_2_ supported on Ti felt for the oxygen evolution reaction (OER). A BPM (SEM in Fig. 2b) under reverse bias was employed with the anion exchange layer (AEL) contacting the anode and the CEL contacting the SC-layer (porous support saturated with electrolyte). The cathode was compressed onto the porous layer, and the anode and cathode flow-field plates sandwiched the system.

The BPM employed in this work sandwiched TiO_2_ nanoparticles as the water dissociation catalyst^17^. This custom BPM can lower the cell voltage by ~1 V compared with commercial BPMs (e.g., Fumasep, details in SI2). The full cell voltage of such custom BPM-based electrolyzers is close to that of anion exchange membrane (AEM)-based systems.

Measurements of the CO_2_/O_2_ ratio in the anode gas stream show that the SC-BPMEA effectively prevents CO_2_ crossover, as required for high SPU (SI1)^8,11^. In agreement with the previous studies^8,11^, the AEM-based MEA (AEMEA) showed an anode CO_2_/O_2_ ratio of ~2 for current densities ranging from 100 to 300 mA cm^−2^ (Fig. 2c). In conventional AEMEAs, the anionic charge carriers are CO_3_^2−^, and thus suffer the loss of one molecule of CO_2_ for every two electrons transferred. The anode CO_2_/O_2_ ratio in the SC-BPMEA (0.06 at 200 mA cm^−2^) is one order of magnitude lower. Control experiments confirm that the CO_2_ detected in the anode is not due to acidification of anolyte (using 0.1 M K_2_SO_4_ instead of 0.1 M KHCO_3_ resulted in a similar CO_2_ /O_2_ ratio, Supplementary Fig. 3). The anode CO_2_/O_2_ ratio decreases as the operating current density increases, an effect we ascribe to an increased flux of protons toward the cathode. This flux decreases the pH at the CEL surface and reduces the diffusion of CO_2_ and HCO_3_^−^/CO_3_^−^ in the CEL^18,19^.

### Impact of the thickness of the SC-layer on CO_2_RR

**Fig. 3:**
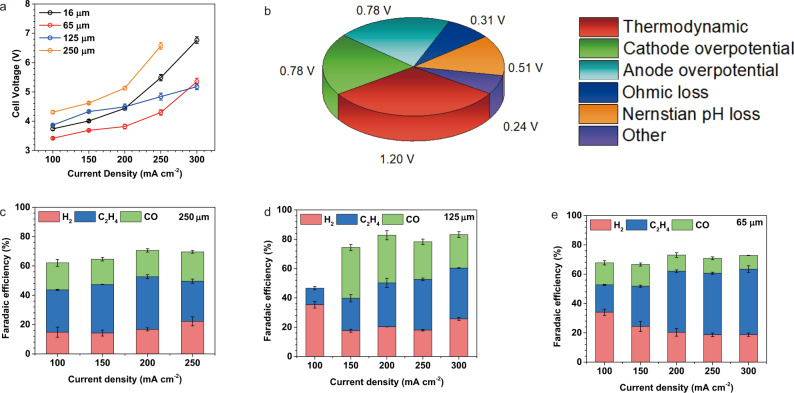
Investigations on the CO_2_RR performance of the SC-BPMEA using catholyte thicknesses of 250, 125, 65, and 16 μm. All the results are collected at 35 °C with a CO_2_ flow rate of 10 sccm cm^−2^ (normalized by the geometric area of the cathode), a catholyte of 0.5 M K_2_SO_4_, and an anolyte of 0.1 M KHCO_3_. **a** The dependence of cell voltages on current density. **b** Distribution of voltage losses measured in the SC-BPMEA with 65 μm thick catholyte operating at 200 mA cm^−2^ (cell voltage = 3.82 V). The breakdown of the voltage is explained in SI7 of the Supplementary Information. The dependence of the CO_2_RR gas products FE on the current density for the SC-BPMEAs with the catholyte thickness of 250 (**c**), 125 (**d**), and 65 μm (**e**). The FE result for 16 μm is shown in Supplementary Fig. 12. The error bars represent the standard deviation of three measurements.

As predicted from simulations, the thickness of the stationary catholyte has a major impact on cell voltage. The cell voltage of the SC-BPMEA decreases as the thickness of the SC-layer decreases (Fig. 3a) from 250 μm (5.1 V, 200 mA cm^−2^) to a minimum at 65 μm (3.8 V, 200 mA cm^−2^). Further thinning the catholyte to 16 μm resulted in higher voltage (4.4 V, 200 mA cm^−2^)—an effect of the lower-porosity support layer used in the 16 μm case (<20% vs. >70% for the thicker layers, see Supplementary Fig. 13a and SI7). A longer ion migration path and higher ohmic resistance partially explain the 0.67 V cell voltage increase as the stationary catholyte thickness increases from 65 to 125 μm. Based on the independently measured ohmic resistance (Supplementary Fig. 13a), increasing the SC-layer thickness from 65 to 125 μm imposes an ohmic voltage increase of merely 0.07 V at 200 mA cm^−2^. Similarly, compared to 65 μm, the 250 μm SC-layer increases the ohmic voltage loss by 0.24 V at 200 mA cm^−2^, while the cell voltage increases by 1.3 V.

The simulations (Fig. 1d) indicate that the thicker SC-layer results in longer transport distances for dissolved CO_2_. The CO_2_ regeneration rate inside the SC-layer also depends on the current density, and for thicker SC-layers (e.g., >125 μm), CO_2_ bubbles are more prone to form near the CEL. These bubbles obstruct ion migration, increasing the ohmic resistance of the SC-BPMEA. Electrochemical impedance spectroscopy measurements (Supplementary Fig. 13d, e) also support this finding. An applied current of 200 mA cm^−2^ resulted in an insignificant change to the high-frequency resistance (HFR) of the SC-BPMEA with a 65 μm-thick SC-layer; while, in contrast, the HFR of the SC-BPMEA with a 125 μm-thick SC-layer increased by 120% after applying 200 mA cm^−2^ for 20 min, leading to a cell voltage 0.6 V higher than for the 65 μm SC-layer.

The cell voltage of the SC-BPMEA with a 65 μm SC-layer operating at 200 mA cm^−2^ is 3.8 V, comparable to the AEM-based neutral-media MEAs operating at similar conditions (difference <±0.05 V)^20–22^. This result demonstrates that the cell voltage of a BPM-based CO_2_RR electrolyzer can be as low as that of an AEM-based electrolyzer with a current density of up to 200 mA cm^−2^, *while suppressing unwanted crossover and providing high SPU*.

Figure 3b shows the breakdown of the 3.8 V cell voltage, determined using methods reported previously^13,21^ (SI7). The factors making up the cell voltage include the thermodynamic potential, cathode overpotential, anode overpotential, ohmic loss, and Nernstian/concentration overpotential (i.e., due to pH gradient)^21^. The sum of these factors accounts for most of the cell voltage, suggesting that the water dissociation overpotential at the AEL/CEL interface of the BPM is small in the SC-BPMEA at 200 mA cm^−2^, in agreement with previous reports employing BPMs fabricated in this way (SI2 and ref. ^17^).

The thickness of the SC-layer also affects selectivity towards CO_2_RR. With thicknesses of 65, 125, and 250 μm, the H_2_ Faradaic efficiencies (FEs) are consistent (~20% at 200 mA cm^−2^, Fig. 3c–e), confirming that high local pH conditions are maintained the cathode in these cases (Fig. 1c). However, reducing the thickness to 16 μm increases the H_2_ FE to 88% at 200 mA cm^−2^ (Supplementary Fig. 14), consistent with a cathodic pH that is reduced due to fast proton transport through a thin SC-layer. Without restricting CO_2_ availability (the performance in Fig. 3 was recorded at a CO_2_ flow rate of 10 sccm cm^−2^), the SC-BPMEAs with the SC-layer thickness of 65, 125, and 250 μm show similar ethylene FE of 35–43%.

### Assessment of SPU in SC-BPMEA

**Fig. 4:**
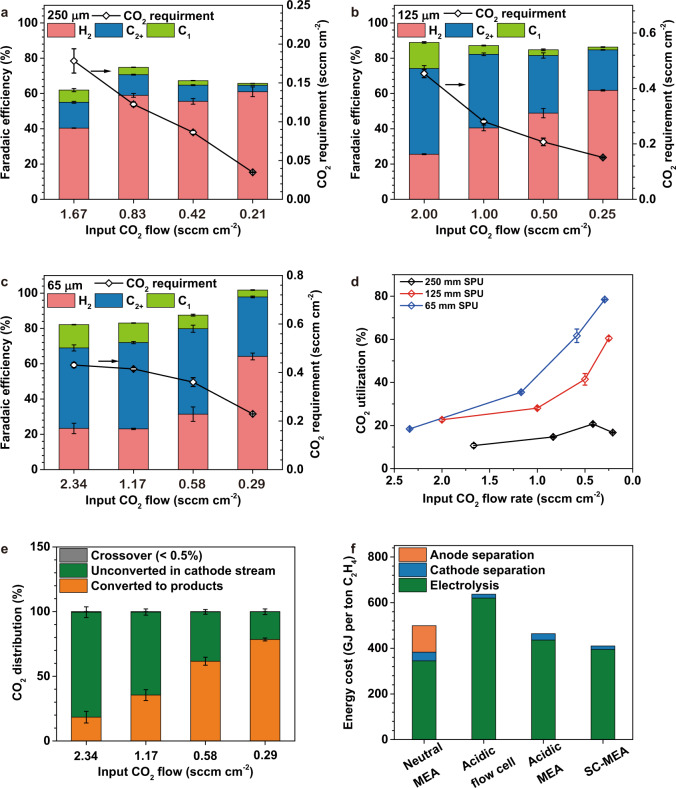
The exploration of the CO_2_RR performance and energy intensity of SC-BPMEA with restricted reactant availability. All the measurements were conducted at 35 °C and 200 mA cm^−2^, and the data were collected after 2 h of continuous operation. **a**–**c** The FE distributions and the CO_2_ requirements (total CO_2_ converted to products) of the SC-BPMEAs with different catholyte thickness and input CO_2_ flow rates (sccm normalized by electrode area). C_1_ refers to CO, formate, and methane. C_2+_ refers to ethylene, ethanol, acetate, and n-propanol. **d** The total CO_2_ single-pass utilization (the CO_2_-to-ethylene single-pass conversion see Supplementary Fig. 13a) for the SC-BPMEAs with different catholyte thickness and input CO_2_ flow rates. **e** Carbon balance in SC-BPMEA with 65 μm 0.5 M K_2_SO_4_ at different input CO_2_ flow rates. See Supplementary Fig. 13b for plots on a logarithmic scale. **f** The comparison of the energy cost distributions among state-of-art CO_2_-to-ethylene electrolyzers. The SC-BPMEA case refers to the input CO_2_ flow rate of 1.17 sccm cm^−2^. The error bars represent the standard deviation of three measurements.

By suppressing the crossover of CO_2_ (e.g., <0.5% of total CO_2_ input at 200 mA cm^−2^, Figs. 2c and 4e), the SC-BPMEA surpasses the SPU of conventional CO_2_-to-C_2+_ electrolyzers, in which carbonate is the dominant charge carrier. Measuring the CO_2_ SPUs with a restricted CO_2_ flow rate is a direct approach to determining the upper bound of SPU in the CO_2_RR electrolyzers^13^.

As the inlet CO_2_ flow rate decreased, the C_2+_ FE of the SC-BPMEA at 200 mA cm^−2^ decreased, accompanied by an increase in the H_2_ FE (Fig. 4a–c). With SC-layer thicknesses of 65 μm (Fig. 4c), as the input CO_2_ flow rate decreases from 1.17 to 0.58 and 0.29 sccm cm^−2^, the C_2+_ FE decreases from 49 to 48% and 34%, while the H_2_ FE increases from 23 to 31% and 64%. This shift is consistent with a CO_2_ mass transport limitation^13,20,21^.

The stationary catholyte thickness affects the SPU of the SC-BPMEA. The SPU gradually increases up to 21, 61, and 78% for the SC-BPMEAs with SC-layer thicknesses of 250, 125, and 65 μm, respectively (Fig. 4d). These results demonstrate that high CO_2_ conversion efficiencies are possible using SC-BPMEAs with SC-layer thicknesses of 125 and 65 μm.

For a given CO_2_ flow rate, a thicker SC-layer produces a lower SPU (Fig. 4d). In the SC-BPMEA, reactant CO_2_ is available from the inlet gas stream and regeneration in the SC-layer. With unrestricted CO_2_ supply (Fig. 3c–e), the H_2_ FEs are similar for different stationary cathode layer thicknesses, indicating that both the CO_2_ availability and local pH are unaffected by catholyte thickness under excess supply conditions. The simulations suggest that the thicker SC-layer results in a lower dissolved CO_2_ flux to the cathode due to the smaller concentration gradient (Fig. 1d). Compared to the SC-BPMEAs with thinner SC-layers, CO_2_ availability with thicker SC-layers decreases more significantly with reducing CO_2_ flow rate, leading to a more dramatic increase in H_2_ FE (Fig. 4a–c).

The experimental trends are generally consistent with those of the simulations. The SC-BPMEA with a dissolved CO_2_ diffusion layer thicker than 75 μm (representing a 250 μm SC-layer) fails to surpass the SPU limit because of insufficient mass transfer. In contrast, a 65 μm SC-layer facilitates efficient mass transport of the regenerated CO_2_ (diffusion layer thickness of 12 μm) and simultaneously promotes high local cathode pH.

As demonstrated in SI10, SC-BPMEAs using acidic and alkaline electrolytes achieve carbon efficiencies comparable to those using neutral electrolytes. The compatibility of SC-BPMEAs with a range of electrolytes offers flexibility in the selection of cathode and anode catalysts. In contrast, acidic CO_2_-to-C_2+_ electrolyzers have only been demonstrated with precious metal anodes^13,14^.

As shown in Supplementary Fig. 18, the SC-BPMEA shows > 50-h stability operating at 200 mA cm^−2^ with limited CO_2_ availability (CO_2_ input flow rate of 1.42 sccm cm^−2^). This operating stability is competitive with that of the neutral-electrolyte-based CO_2_-to-C_2+_ electrolyzers^23,24^.

### Can a cation-exchange membrane replace the BPM in SC-BPMEA?

**Fig. 5:**
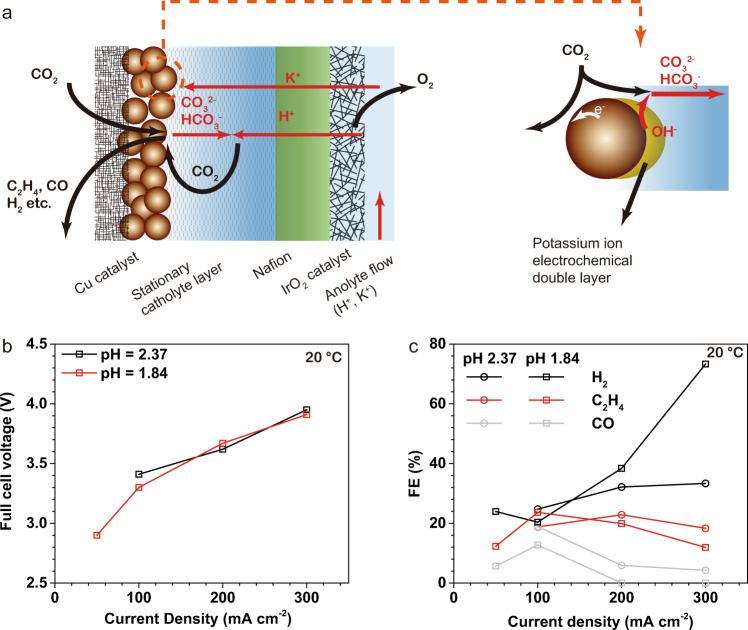
Investigations on the CO_2_RR performance of the SC-CEMEA. The stationary catholyte is 0.5 M K_2_SO_4_ and the anolyte is 0.5 M K_2_SO_4_ plus 0.1 (pH = 2.37) or 0.5 M (pH = 1.84) H_3_PO_4_. All the measurements were performed at 20 °C and a CO_2_ flow rate of 10 sccm cm^−2^. **a** The scheme of the SC-CEMEA. **b** Full cell voltages at various current densities. **c** The gas product FEs at various current densities.

We attempted to extend the SC-layer strategy in a CEM-based MEA cell (i.e., SC-CEMEA, Fig. 5a) using an acidic anolyte with pH < 2.4, expecting a lower cell voltage than the SC-BPMEA while maintaining high SPU. We found that in the SC-CEMEA, the CO_2_ crossover was essentially eliminated. This observation is ascribed to the lower pH near the stationary catholyte layer/CEM interface, as shown in Fig. 5a.

SC-CEMEA shows a lower full cell voltage (Fig. 5b) compared to the SC-BPMEA presented, partly due to the lower resistance of the CEM and the absence of water dissociation overpotential. Meanwhile, it has a reasonable CO_2_RR selectivity over HER (Fig. 5c) due to the cation effect and high local pH induced by the presence K^+^ in the SC-layer (Fig. 5a). However, this design is not amenable to steady-state operation without continuous addition of acid and salt to the anolyte, as the initial pH gradient will be eliminated due to co-ion transport and neutralization. We found the CO_2_RR selectivity decreases over time and approaches 100% H_2_ after ~3 h.

We also observed that the SC-CEMEA design periodically ejects electrolyte from the cathode flow channel, likely due to poor water balance. On the anode, the OER generates one proton per one electron transfer. The charge carriers across the CEM are primarily H^+^, although neutral ion pairs will diffuse as well. At the cathode K^+^ makes up the electrochemical double layer at the Cu surface, and the steady-state K^+^ profiles are governed by the electric and chemical-potential gradients that develop under operation, which usually takes tens of seconds^19^. H^+^ migrates to the cathode and combines with OH^−^ (or CO_3_^2−^/HCO_3_^−^), producing water at the cathode. The protons also drag water molecules (~1 per proton)^25^ by electro-osmosis. We accordingly calculate the water balance for different cathode products as listed in Table 2. The water generated and transported to the cathode appears to dilute and push out the electrolyte in the stationary catholyte layer, of which the volume is small (ca. 10 μL per cm^2^ electrode area). This phenomenon results in flooding of the cathode (as confirmed experimentally) and loss of supporting electrolyte, thus degrading performance. In the BPMEA design, it is likely that the BPM slows co-ion transit across the membrane, compared to the CEM, by the large outward flux of OH^−^ and H^+^ from the water dissociating junction.

**Table 2:** The cathode-anode water balance in an SC-CEMEA.

Product (1 mol)	Water balance in the cathode (mol)				Water balance in the anode (mol)
	Consumed	Generated	Dragged in	Net	Consumed and dragged out
CO	1	2	2	+3	3
HCOO^−^	1	1	1	+1	2
CH_3_COO^−^	5	7	7	+9	11
C_2_H_4_	8	12	12	+16	18
C_2_H_5_OH	9	12	12	+15	18
CH_4_	6	8	8	+10	12
H_2_	2	2	2	+2	3

### Energy assessment of the SC-BPMEA with optimal SC-layer

The energy costs (measured in gigajoules per tonne of the target product, GJ/t) for a CO_2_-to-C_2+_ electrolyzer include the electrolysis electrical energy, cathodic stream separation, and anodic stream separation^13^. CO_2_RR performance metrics of importance include cell voltage, target product FE, SPU, and CO_2_ crossover^5^. High SPU and high energy efficiency have not been accomplished simultaneously in C_2+_ electroproduction. In SC-BPMEAs, a higher SPU reduces the energy required for cathode separation, but the accompanying decrease in the ethylene selectivity (Fig. 4c) elevates the specific energy requirement^5^. We carried out a total energy assessment of the SC-BPMEA and other state-of-art CO_2_-to-ethylene electrolyzers and summarized the results in Table 3, Fig. 4f, and Supplementary Table 2.

**Table 3:** Comparison of the energy intensity between various CO_2_-to-ethylene electrolyzers and this work.

Metrics	Neutral-MEA^a^^21^	Acidic flow cell^a13^	Acidic MEA^a14^	This work (10 sccm cm^−2^)	This work (1.17 sccm cm^−2^)
Cell type	MEA	Flow cell	MEA	SC-BPMEA	SC-BPMEA
Electrolyte	Neutral	Acidic	Acidic	Neutral	Neutral
Full cell voltage (V)	3.75	4.20	3.80	3.82	3.82
Ethylene FE (%)	45	28	36	42	40
Current density (mA cm^−2^)	150	1200	100	200	200
Input CO_2_ flow rate (sccm cm^−2^)	8	3	0.8	10	1.17
Total CO_2_ SPU (%)	3	78	34	4.1	35
CO_2_-to-ethylene (%)	1.2	28	7.6	2.0	17
Demonstrated stability (hours)	100	14	12	52	
Energy intensity (GJ per ton ethylene)					
Electrolyzer electricity	345	620	436	385	395
Cathode separation	38	17	28	85	15
Anode separation	116	0^b^	0^b^	0^b^	0^b^
Overall energy	499	637	465	470	410

^a^The energy intensities of reference CO_2_-to-ethylene devices operating under the reported conditions are calculated, and those that provide the lowest energy intensity are presented in this table.

^b^Crossover of CO_2_ in the acidic flow cell, acidic MEA, and SC-BPMEA are each lower than 0.5% of input CO_2_. Therefore, we assume the anodic separation energy to be 0.

All the energy costs are normalized per ton of ethylene produced. The energy intensities of the SC-BPMEA operating at other CO_2_ input flow rates are listed in Table S3 of Supplementary Information.

The energy consumption of an alkaline CO_2_RR electrolyzer^2^ is listed in Supplementary Table 2. In such systems, CO_2_ and OH^−^ react to form carbonate continuously. This carbonate has to be recovered to maintain the CO_2_RR performance of such a system, consuming 5.5 GJ per tonne CO_2_^5^. In the alkaline CO_2_RR electrolyzers, ca. 63 tonne of CO_2_ transforms to carbonate to produce 1 tonne of ethylene, representing an energy penalty of 350 GJ^5^. This costs at least $1900 per tonne of ethylene, while its market price is $800–1000 per tonne^26^. The alkaline electrolyzers thus do not allow for ethylene electrochemical production to be yet profitable.

In neutral-media CO_2_RR electrolyzers, recovering the CO_2_ from the anodic gas stream results in significant energy costs. In the context of highly selective conversion (i.e., CO_2_-to-ethylene with unity selectivity), the recovery process requires an energy input of 52 GJ to produce every tonne of product. In practice, due to non-unity product selectivity, the process is even more prohibitive, i.e., requiring an energy penalty of 80–130 GJ for producing one tonne of ethylene^5^.

As the SPU increases from 4 to 35%, we found a dramatic decrease in energy associated with cathode separation—from 85 to 15 GJ/t ethylene (Table 3), with the ethylene FE reduced by only 2%. Further increasing the SPU beyond 35% does not substantially reduce the energy cost associated with cathodic separation (Supplementary Table 2). This finding agrees with a recent energy analysis that in a (bi)carbonate-free CO_2_-to-C_2+_ electrolyzer, improving SPU over 40% offers an insignificant benefit to the downstream separation cost^4^. Pursuing an SPU > 35% decreases ethylene FE by more than 4% when using the SC-BPMEA, and thus the increased input electricity cost exceeds the savings in the cathodic separation (Table 3 and Supplementary Table 2). Therefore, 35% SPU is the most favourable condition for the present SC-BPMEA.

The energy intensity of producing ethylene in SC-BPMEA is ~30% lower than that in conventional neutral-electrolyte-based CO_2_ electrolyzers (Fig. 4f and Table 3). In conventional neutral-electrolyte CO_2_-to-ethylene electrolyzers, the CO_2_ crossover (at least 70%)^21^ costs 60–90 GJ per ton of ethylene to recover CO_2_ from the anodic O_2_ stream^5^. Notably, this energy penalty cannot readily be reduced, independent of optimizing catalysts and operating conditions (e.g., input CO_2_ flow rates, reaction rates, operating temperature, and pressure)^6^. In contrast, crossover CO_2_ in SC-BPMEA is < 0.5% of the total CO_2_ input, minimizing the energy cost of anodic separation.

Recently, CO_2_-to-ethylene conversion has been achieved in acidic electrolytes in both flow cell^13^ and MEA configurations^14^. These systems enabled CO_2_ SPUs exceeding 75% and also mitigated the energy cost associated with anodic separation (Table 3). Owing to the strongly acidic environment, the flow cell enables an ethylene FE of 28% at a full-cell potential of 4.2 V. The acidic MEA used an anion-exchange ionomer coating on the catalyst layer to promote CO_2_RR over HER. The modification of the surface with the anion exchange ionomer resulted in a higher ohmic loss, and thus the cell required potentials of 3.8 and 4.4 V at 100 and 200 mA cm^−2^, respectively. These devices thus eliminated the anodic CO_2_/O_2_ separation energy but at the penalty of larger cell voltages and/or lower ethylene FEs. In contrast, SC-BPMEA shows a cell voltage of 3.8 V at 200 mA cm^−2^ with an ethylene FE of 42%—voltages and selectivities comparable to the best conventional neutral-electrolyte CO_2_-to-ethylene MEAs^21^. Compared to acidic systems, the energy intensity of the SC-BPMEA is 36% and 12% lower than acidic flow cell and acidic MEA, respectively (Fig. 4f and Table 3).

## Discussion

We demonstrate a BPM-based CO_2_-to-C_2+_ MEA, with a judiciously-designed SC-layer between catalyst and BPM, that overcomes the (bi)carbonate-formation reactant loss issue without compromising performance. The composition and thickness of the SC-layer determine the CO_2_RR performance and SPU via a strong influence on the local pH and the chemistry and transport of CO_2._ The buffering capacity and the thickness of the SC-layer determine the efficiency of the regeneration, the transport, and the availability of reactant CO_2_. These effects were predicted in simulations and supported by experiments. The SC-BPMEA design largely eliminates the energy penalty associated with the CO_2_ loss in electrochemical CO_2_ reduction.

The performance of the SC-BPMEA might be further improved using, for example, ionic liquid or other organic salts as the catholyte, and by optimizing the porosity, structure, and hydrophobicity of the porous support layers. The CO_2_RR performance of the SC-BPMEA might be improved with new cathodic catalysts, optimizing the loading and processing of the catalyst layer, and by implementing BPMs with further-lowered water-dissociation voltage loss. Broadly, the SC-BPMEA is a useful platform for evaluating CO_2_RR catalysts operating with high CO_2_ utilization. The strategy and findings presented here are also relevant to the electrochemical systems such as nitrate reduction and (bi)carbonate reduction, where controlling dissimilar microenvironments near each electrode is useful, and the exchange/transport of species (other than OH^−^ or H^+^) between cathode and anode is problematic.

## Methods

### Materials

Phosphoric acid (H_3_PO_4_, 85%), potassium sulfate (K_2_SO_4_, 99%), potassium bicarbonate (KHCO_3_, 99.7%), potassium chloride (KCl, 99%), potassium hydroxide (KOH, 99.95%), copper nanoparticles (25 nm), Nafion^TM^ 1100 W (5 wt. % in a mixture of lower aliphatic alcohols and water) and isopropanol (IPA, 99%) were purchased from Sigma Aldrich and used as received. Titanium oxide nanoparticles (TiO_2_, Aeroxide P25) were purchased from Fisher Scientific and used as received. The porous supports were also purchased from Fisher Scientific: 125 μm PVDF (0.45 μm pore size), 65 μm PTFE (0.44 μm pore size) and 16 μm PC (0.4 μm pore size). Nafion^TM^ 212, Nafion^TM^ XL, Fumasep (FAS-PET-130) and titanium (Ti) felt were purchased from Fuel Cell Store. Iridium(IV) chloride hydrate (Premion^®^, 99.99%, metals basis, Ir 73% min) was purchased from Alfa Aesar. The water used in this study was 18 MΩ Milli-Q deionized- (DI-) water. Nafion membranes were activated through the following procedure: 1 h in 80 °C 1 M H_2_SO_4_—1 h in 80 °C H_2_O_2_—1 h in 1 M H_2_SO_4_—stored in DI-water. Fumasep was used as received and stored in 1 M KCl. Piperion (40 μm) was purchased from W7Energy and stored in 0.5 M KOH.

### Fabrication of water dissociation catalyst layer of the custom bipolar membrane (BPM)

The water dissociation catalyst layer was fabricated following a similar procedure in a previous report^17^. TiO_2_ nanoparticles inks were prepared by sonicating the mixture of TiO_2_, DI-water, and IPA with the weight ratio of 1: 833: 2833 for 30 min. TiO_2_ nanoparticle ink was spray-coated onto a Nafion 212 membrane, of which the edges were sealed by Kapton tape. The exposed membrane dimension was 2.2 cm × 2.2 cm. The nominal loading of TiO_2_ is 0.2 mg cm^−2^. The TiO_2_-coated Nafion^TM^ was immediately used for assembling electrolyzers once prepared.

### Electrode preparation

For the CO_2_RR, we prepared the gas diffusion electrodes (GDEs) by spray-depositing a catalyst ink dispersing 1 mg mL^−1^ of Cu nanoparticles and 0.25 mg mL^−1^ of Nafion^TM^ 1100 W in methanol onto a hydrophobic carbon paper. The mass loading of Cu NPs in the GDE was kept at 1.5 mg/cm^2^. The GDEs were dried in the air overnight prior to experiments.

The OER electrode preparation procedure involves: etching the Ti felt in hydrochloric acid at 70 °C for 40 min; rinsing the etched Ti felt with DI water; immersing the Ti felt into an Ir(IV) chloride hydrate solution; drying and sintering the Ir-loaded Ti felt. The loading, drying, and sintering steps were repeated until a final Ir loading of 1.5 mg cm^−2^ was achieved.

### Assembly of the stationary catholyte membrane electrode assembly (SC-BPMEA)

The MEA set (5 cm^2^) was purchased from Dioxide Materials. A cathode was cut into a 2.1 cm × 2.1 cm piece and placed onto the MEA cathode plate with a flow window with a dimension of 2.2 cm × 2.2 cm. The four edges of the cathode were sealed by Kapton tape, which also made the flow window fully covered. The exposed cathode area was measured every time before the electrochemical tests, in the range of 3.1 to 4.2 cm^2^. Onto the cathode, a porous support layer (2 cm × 2 cm with various thicknesses, 250 μm was stacking two 125 μm-thick PVDF) saturated with desirable electrolyte (sonicated in electrolyte for 15 min to degas) was carefully placed. This porous support layer serves as the ‘stationary catholyte layer (SC-layer).’ The considerations of membrane selection can be found in SI2 and SI4 of the Supplementary Information. When using the custom BPM, a TiO_2_-coated Nafion membrane was placed onto the SC-layer with the TiO_2_ layer facing up, then covered by a Piperion (5 cm × 5 cm) membrane. When using Fumasep BPM, the membrane was placed with its cation-exchange layer (CEL) facing the cathode side. An IrO_2_ loaded Ti felt (2 cm × 2 cm) was placed onto the anion-exchange layer (AEL) of the BPM.

### Scanning electron microscopy (SEM)

Images of cathode and custom BPM were captured by an FEI Quanta FEG 250 environmental SEM.

### Electrochemical measurements

Throughout all experiments, CO_2_ flowed to the cathode side at 10 sccm cm^−2^ unless otherwise specified, while the anode side was fed with neutral 0.1 M KHCO_3_ at 10 mL/min by a peristaltic pump unless otherwise specified. The electrochemical measurements were performed with a potentiostat (Autolab PGSTAT204 with 10A booster). The cell voltages reported in this work are not iR corrected. The system was allowed to stabilize at the specific conditions for > 1000 s before recording the results. All the error bars represent standard deviations based on three measurements.

### Product analysis

The CO_2_RR gas products, oxygen, and CO_2_ were analyzed by injecting the gas samples into a gas chromatograph (Perkin Elmer Clarus 590) coupled with a thermal conductivity detector and a flame ionization detector. The gas chromatograph was equipped with a Molecular Sieve 5A Capillary Column and a packed Carboxen-1000 Column with argon as the carrier gas. The volumetric gas flow rates in and out of the cell were measured with a bubble column. The FE of a gas product is calculated as follows:

1 $${{{{{{\rm{FE}}}}}}}_{i}={x}_{i}\times \frac{{VP}}{{RT}}\times \frac{{n}_{i}F}{J}$$

Where *x*_*i*_ is the volume fraction of the gas product *i*, *V* is the outlet gas flow rate in L s^−1^, *P* is atmosphere pressure 101.325 kPa, *R* is the ideal gas constant 8.314 J mol^−1^ K^−1^, *T* is the room temperature in K, *n*_*i*_ is the number of electrons required to produce one molecule of product F is the Faraday Constant 96485 C mol^−1^, and *J* is the total current in A.

The liquid products from the cathode side of the SC-BPMEA were collected using a cold trap cooled to 0 °C. The collected liquid was combined with anolyte (some crossover liquid product) for quantifying by the proton nuclear magnetic resonance spectroscopy (^1^H NMR) on an Agilent DD2 500 spectrometer in D_2_O using water suppression mode and dimethyl sulfoxide (DMSO) as the internal standard. For each plot of liquid product quantification, fresh anolyte was used, and the duration of the collection was 30 min. The FE of a liquid product is calculated as follows:

2 $${{{{{{\rm{FE}}}}}}}_{i}={m}_{i}\times \frac{{n}_{i}F}{{Jt}}$$

Where *m*_*i*_ is the quantity of the liquid product *i* in mole, *t* is the duration of product collection (1800 s).

The CO_2_ SPU calculation is detailed in SI1 of Supplementary Information.

### COMSOL one-dimensional modelling

**Fig. 6:**
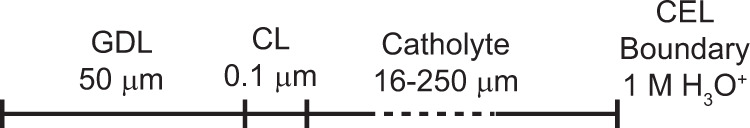
The schematic of 1D COMSOL modelling. GDL and CL refer to gas diffusion layer and catalyst layer, respectively.

The electrochemical reaction model was performed by COMSOL Multiphysics version 5.5. This simulation was built upon previous modelling work^27–30^. The local pH and different species concentrations were simulated for different catholyte thicknesses (16, 65, 125, and 250 μm). Two different catholytes (K_2_SO_4_ and KHCO_3_) were used in the simulation. All the chemical reactions between species were considered in this one-dimensional modelling. The simulation (Fig. 6) included a 50 μm thick gas diffusion layer (GDL), a 0.1 μm thick Cu cathode catalyst (CL), a catholyte region with various thicknesses indicated above, and a cation exchange layer (CEL) boundary.

Constant concentration (Dirichlet) boundary conditions were used. Specifically, a constant concentration 37.8 mM of CO_2_ was assumed within the GDL layer, as this region is in direct contact with the input CO_2_ flow and thus assumed to be at equilibrium with gas phase CO_2_ over this region for the purposes of the simulation. The BPM was interpreted as a boundary with a constant species concentration (1 M H_3_O^+^ at the CEL surface)^18,31^, because it was assumed to generate protons as the dominant ionic charge carrier at a constant rate under constant current density (200 mA cm^−2^).

A user-controlled mesh is employed in the COMSOL simulation. Edge type of mesh is used for GDL, CL, catholytes, respectively. Specifically, the mesh distribution is predefined with an interval of 500 nm for GDL and catholytes, and an interval of 5 nm for CL.

Five different electrode reactions were considered at the cathode catalyst layer in this simulation. Specifically, the hydrogen evolution reaction and CO_2_ reduction reactions to CO, CH_4_, C_2_H_4,_ and C_2_H_5_OH occurred at the cathode catalyst layer. In SC-BPMEA, the catalyst layer is immersed in a catholyte. Thus the simulation considers no gas-phase transport in the catalyst layer. The carbonate equilibrium reactions, corresponding catholyte buffer reactions, and a water dissociation reaction were considered in the catholyte region. The electrochemical reaction rates of the specific products were determined from experimental results. They are calculated based on the same manner as previous work^17^.

The electrochemical reactions at cathode catalyst layer:

$${2{{{{{\rm{H}}}}}}}_{2}{{{{{\rm{O}}}}}}+2{e}^{-}\to {{{{{\rm{H}}}}}}_{2}+2{{{{{\rm{O}}}}}}{{{{{\rm{H}}}}}}^{-}$$

$${{{{{\rm{CO}}}}}}_{2}+{{{{{\rm{H}}}}}}_{2}{{{{{\rm{O}}}}}}+2{e}^{-}\,\to {{{{{\rm{CO}}}}}}+2{{{{{\rm{OH}}}}}}^{-}$$

$${{{{{\rm{CO}}}}}}_{2}+{{{{{\rm{H}}}}}}_{2}{{{{{\rm{O}}}}}}+8{e}^{-}\,\to {{{{{\rm{CH}}}}}}_{4}+8{{{{{\rm{OH}}}}}}^{-}$$

$$2{{{{{\rm{CO}}}}}}_{2}+{8{{{{{\rm{H}}}}}}}_{2}{{{{{\rm{O}}}}}}+12{e}^{-}\,\to {{{{{\rm{C}}}}}}_{2}{{{{{\rm{H}}}}}}_{4}+12{{{{{\rm{OH}}}}}}^{-}$$

$$2{{{{{\rm{CO}}}}}}_{2}+{9{{{{{\rm{H}}}}}}}_{2}{{{{{\rm{O}}}}}}+12{e}^{-}\,\to {{{{{\rm{C}}}}}}_{2}{{{{{\rm{H}}}}}}_{5}{{{{{\rm{OH}}}}}}+12{{{{{\rm{OH}}}}}}^{-}$$

The heterogenous electrochemical reaction rates are determined by the following equations:

3 $${r}_{i}=\frac{{I}_{i}}{{n}_{i}F}\;*\; \frac{\varepsilon }{{L}_{{{{{{\rm{catalyst}}}}}}}}$$

4 $${r}_{{{{{{{\rm{CO}}}}}}}_{2}}=-\frac{{I}_{{{{{{\rm{total}}}}}}}}{F}\left(\frac{{{{{{{\rm{FE}}}}}}}_{{{{{{\rm{CO}}}}}}}}{2}+\frac{{{{{{{\rm{FE}}}}}}}_{{{{{{\rm{CH}}}}}}4}}{8}+\frac{{{{{{{\rm{FE}}}}}}}_{{{{{\rm{C2H4}}}}}}}{12}+\frac{{{{{{{\rm{FE}}}}}}}_{{{{{\rm{C2H5OH}}}}}}}{12}\right)* \frac{\varepsilon }{{L}_{{{{{{\rm{catalyst}}}}}}}}$$

5 $${r}_{{{{{{{\rm{OH}}}}}}}^{-}}=\frac{{I}_{{{{{{\rm{total}}}}}}}}{F}\;*\; \frac{\varepsilon }{{L}_{{{{{{\rm{catalyst}}}}}}}}$$

Where *I*_*i*_ represents the partial current density for CO, CH_4_, C_2_H_4,_ and C_2_H_5_OH occurred at the cathode catalyst layer, respectively. *n*_*i*_ represents the number of electrons transferred per mole reactant. *F* represents faraday’s constant. *I*_total_ represents the total current density. The FEs for the specific product is determined by the experimental results shown in Fig. 3e. *ε* represents the catalyst porosity value. *L*_catalyst_ represents the cathode catalyst length.

The chemical reactions at the catholyte region and the corresponding forward *k*_f_ rate constants and reverse *k*_r_ rate constants taken from the literature^32^ (see Supplementary Table 3).

The *Transport of Diluted Species* physics model was used. The Nernst-Planck set of equations governed the species diffusion, and they were calculated in the same manner as previous work^13,14^. Migration was ignored for simplicity as the experiments were performed in the concentrated electrolyte. The ion species transport is thus calculated by solving the two equations below.

6 $$\frac{\partial {c}_{i}}{\partial t}\;+\;\frac{\partial {J}_{i}}{\partial x}\;+\;{r}_{i}={R}_{i}$$

7 $${J}_{i}=-\frac{{D}_{i}\partial {c}_{i}}{\partial x}$$

8 $${D}_{i}=\frac{{\varepsilon }_{p}}{{\tau }_{F,i}}\;*\; {D}_{F,i}$$

9 $${\tau }_{F,i}={\varepsilon }_{p}^{-1/3}$$

Where *J*_*i*_ is the molar flux, and *r*_*i*_ represents the heterogeneous electrode reactions for CO_2_ reduction that were modelled at the cathode catalyst layer. *R*_i_ represents the rates of the homogeneous reactions indicated above. The Millington and Quirk model is used to determine the effective diffusivity, *D*_*i*_. *ε*_*p*_ represents porosity coefficient. *τ*_*F,i*_ represents tortuosity coefficient.

The porosity value of 0.6 was used for the cathode catalyst and the porosity value of 1 for the catholyte region. The species diffusion coefficients are listed in Supplementary Table 4^33–36^.

Henry’s law and sets of Sechenov equation are applied to calculate the CO_2_ concentration. The concentration of CO_2_ in electrolytes depends on temperature and pressure. It is estimated in the same manner as previous work^13,14^. The Sechenov coefficients are listed in Supplementary Table 5^37^.

### Energy assessment

We evaluated the energy consumptions for electrolyzer electricity, cathodic separation, and anodic separation in the context of ethylene. We consider the state-of-the-art CO_2_RR systems from the literature, including alkaline flow-cell electrolyzers, neutral MEA electrolyzers, acidic flow-cells, and MEAs. This consideration is based on the performance metrics, including selectivity, productivity, and full-cell voltage—the combination reflects as energy intensity of producing multi-carbon products (i.e., ethylene). The proximity of these performance metrics will help refine the effect of anodic and cathodic separation on the energy requirement for producing ethylene. We summarize the input parameters to the model for all the systems. The energy assessment model, as well as the assumptions, are based on the previous work^5^. Ideally, it will be interesting to use experimental/modelling data corresponding to the exact gas composition from the CO_2_-to-C_2+_ device. However, at present, there is a gap in published literature. We, therefore, employed one of the most widely used models^38^ (i.e., biogas upgrading) as the best approximation for evaluating the energy cost associated with cathode gas separation. The details of calculations for the carbon regeneration (for alkaline flow cell) and cathodic separation (for all the electrolyzers), can be found in previous work^26^. The anodic separation (for neutral MEA electrolyzer) is modelled based on an alkaline capture solvent^39^. The amount of CO_2_ crossover to the anode is calculated for one tonne of ethylene produced. The energy required to separate the CO_2_/O_2_ mixture is calculated based on a recent report by Carbon Engineering^40^, in which 5.25 GJ/tonne CO_2_ thermal energy and 77 kWh/tonne CO_2_ are reported to be required to capture CO_2_ and release at 1 bar. This energy consumption is a typical value for the alkaline capture process^39^. For acidic flow-cell and MEA electrolyzers, we assume no energy cost associated with the anodic separation considering no CO_2_ availability at the anodic gas stream^13^.

## Supplementary information

Supplementary Information

## Data Availability

All the data generated in this study are provided in the Supplementary Information and in the Source Data file. Source data are provided with this paper.

## Source data

Source Data
