# Supplementary material for: Bipolar membrane electrolyzers enable high single-pass CO2 electroreduction to multicarbon products
Source: Nat Commun. 2022 Jun 24;13:3609. doi: 10.1038/s41467-022-31295-3 (PMC9232613; doi:10.1038/s41467-022-31295-3)
Supplement: Supplementary file 1 — Supplementary Information [file 41467_2022_31295_MOESM1_ESM.docx]

**Supplementary Information**

**Bipolar membrane electrolyzers enable high single-pass CO_2_ electroreduction to multicarbon products**

Ke Xie,^1,†^ Rui Kai Miao,^2, †^ Adnan Ozden,^2^ Shijie Liu,^2^ Zhu Chen,^1^ Cao-Thang Dinh,^3^ Jianan Erick Huang,^1^ Qiucheng Xu,^4^ Christine M. Gabardo,^2^ Geonhui Lee,^1^ Jonathan P. Edwards,^2^ Colin P. O’Brien,^2^ Shannon W. Boettcher,^4^ David Sinton,^*2^ Edward H. Sargent^*1^

^1^Department of Electrical and Computer Engineering, University of Toronto, 10 King’s College Road, Toronto, ON M5S 3G4, Canada

^2^Department of Mechanical and Industrial Engineering, University of Toronto, 5 King’s College Road, Toronto, ON M5S 3G8, Canada

^3^Department of Chemical Engineering, Queen’s University, 19 Division Street, Kingston, Ontario K7L 3N6, Canada

^4^Department of Chemistry and Biochemistry, University of Oregon, Eugene, OR 97403, USA.

^†^These authors contributed equally

^*^Correspondence:

ted.sargent@utoronto.ca (E.H.S.)

sinton@mie.utoronto.ca (D.S.)

**Table of Contents**

| **SI1 Analysis and comparison of CO_2_ single-pass utilization among state-of-art CO_2_RR electrolyzers.** | **3** |
| --- | --- |
| **SI2 Analysis of BPM functions** | **6** |
| **SI3 Investigating the feasibility of the existing BPM-based MEAs for high carbon utilization in CO_2_-to-C_2+_** | **9** |
| **SI4 Additional numerical simulation results on non-buffer stationary catholyte layer.** | **13** |
| **SI5 The use of buffer catholyte in the stationary catholyte layer.** | **14** |
| **SI6 Exploration of the operating conditions for SC-BPMEA with non-buffer catholyte.** | **17** |
| **SI7 Analysis on the cell voltage of SC-BPMEA operating at 35 ^o^C, 200 mA cm^-2^, with KHCO_3_ anolyte and 65 μm 0.5M K_2_SO_4_ catholyte layer.** | **20** |
| **SI8 The FE distributions of the SC-BPMEA with 16 μm 0.5 M K_2_SO_4_ SC-layer.** | **23** |
| **SI9 Additional CO_2_ SPU results for SC-BPMEAs with different SC-layer thickness.** | **24** |
| **SI10 The compatibility of SC-BPMEA with acidic and basic anolytes.** | **25** |
| **SI11 Stability of SC-BPMEA with 65 μm 0.5 M K_2_SO_4_ and restricted CO_2_ flow rate** | **27** |
| **SI12 Energy assessments of the SC-BPMEA operating at different CO_2_ input flow rates.** | **28** |
| **SI13 Additional information for COMSOL one-dimensional modeling** | **29** |
| **References** | **30** |

**SI1 Analysis and comparison of CO_2_ single-pass utilization among state-of-art CO_2_RR electrolyzers.**

For CO_2_RR, a high local pH (>11) near the cathode is required to suppress the competing hydrogen evolution reaction (HER) and to enhance the selectivity towards C_2+_ products.^1–6^ To maintain such a high local pH, present-day CO_2_RR electrolyzers typically use a flowing alkaline electrolyte reservoir.^1,2,7^ For the same reason, MEAs typically use alkaline anion-exchange membranes (AEMs) and anolytes.^18^ However, locally alkaline conditions absorb CO_2_:

| CO_2_ + 2OH^-^ → CO_3_^2-^ + H_2_O | [1] |
| --- | --- |
| CO_2_ + OH^-^ → HCO_3_^-^ | [2] |

Meanwhile, the major cathode reactions in neutral or alkaline media include:

| CO_2_ + H_2_O + 2e^-^ → CO + 2OH^-^ | [3] |
| --- | --- |
| CO_2_ + H_2_O + 2e^-^ → HCOO^-^ + OH^-^ | [4] |
| 2CO_2_ + 5H_2_O + 8e^-^ → CH_3_COO^-^ + 7OH^-^ | [5] |
| 2CO_2_ + 8H_2_O + 12e^-^ → C_2_H_4_ + 12OH^-^ | [6] |
| 2CO_2_ + 9H_2_O + 12e^-^ → C_2_H_5_OH + 12OH^-^ | [7] |
| CO_2_ + 6H_2_O + 8e^-^ → CH_4_ + 8OH^-^ | [8] |
| 3CO_2_ + 13H_2_O + 18e^-^ → C_3_H_7_OH + 18OH^-^ | [9] |
| 2H_2_O + 2e^-^ → H_2_ + 2OH^-^ | [10] |

All these reactions generate hydroxide, of which the rate (in mole per second, *M_OH_*) is:

| $M_{OH}=\sum_{i}^{n} \frac{J\times{FE}_{[i]}\times k_{[i]}}{F}$ | (1) |
| --- | --- |

where *J* is the current in amps, *FE_[i]_* is the faradaic efficiency of the specific reaction [3-10], *F* is the Faraday constant, and *k_[i]_* is the number of OH^-^ generated per electron transferred in the specific reaction [3-10]. For the cathode reactions [4] and [5], the *k_[i]_* values are 0.5 and 0.875, respectively; for the other cathode reactions the *k_[i]_* values are 1. The configuration A and B of Table 1 in the main text show that, in neutral media, the in-situ generated hydroxide reacts with CO_2_ to form carbonate and bicarbonate, which migrate to anode, combine with protons (generated by the oxygen evolution reaction), and release CO_2_ into anode gas stream. This phenomenon is known as CO_2_ crossover.^10–12^ Therefore, the CO_2_/O_2_ ratio in anode gas provides insight into the identity of the anionic charge carrier(s) that combine with the H^+^ generated on the anode.^13^ Ideally, if the charge carrier is HCO_3_^-^ or CO_3_^2-^, the CO_2_/O_2_ ratio in the anode gas stream is 4 or 2, respectively.^13^ While the other charge carriers like OH^-^, HCOO^-^ or CH_3_COO^-^ do not release CO_2_ by acidification at the anode, but do lead to loss of product.

Based on the analysis above, the inlet CO_2_ (*C_in_*) is balanced by four parts: the CO_2_ in outstream (*C_1_*), the electrochemically reduced CO_2_ (*C_2­_*), the absorbed CO_2_ (*C_3_*), and the crossover CO_2_ (*C_4_*). In other words, the mass balance of CO_2_ (in mole per second) is:

| $C_{in}=C_{1}+C_{2}+C_{3}+C_{4}$ | (2) |
| --- | --- |

The carbon utilization efficiency is evaluated by single-pass utilization (SPU):

| $SPU=\frac{C_{2}}{C_{in}}$ | (3) |
| --- | --- |

In conventional flow cells and MEAs, some studies have demonstrated that *C_1_* can be negligible compared to *C_2_* and *C_4_* by carefully tuning *C_in_*. When the CO_2_ absorption in the system reaches a steady-state, *C_3_* is almost zero. Therefore, the upper limit of SPU is:

| $SPU_{limit}=\frac{C_{2}}{C_{2}+C_{4}}$ | (4) |
| --- | --- |
| $C_{2}=\sum_{i}^{n} \frac{J\times{FE}_{[i]}}{n_{[i]}F}$ | (5) |

where *J* is the current in amps, *FE_[i]_* is the faradaic efficiency of the specific reaction [3-9], *n_[i]_* is the number of electrons transferred per consumed CO_2_ in the specific reaction [3-9], and *F* is the Faraday constant. In neutral media, *C_4_* ranges from 0.5 to 1 times *M_OH_*, depending on the species of charge carrier crossing over the AEM. To evaluate the upper limit of the SPU, *C_4_ = 0.5 M_OH_*. Substituting (1) and (5) into (4) gives:

| $SPU_{limit}=\frac{\sum_{i}^{n} \frac{{FE}_{[i]}}{n_{[i]}}}{\sum_{i}^{n} {FE}_{[i]}\times(\frac{1}{n_{\left[ i \right]}}+0.5k_{\left[ i \right]})}$ | (6) |
| --- | --- |

Therefore, in the conventional flow cells and MEAs operating in neutral media, the upper limits of SPU depend on their product distributions. For example, the SPU upper limits of the systems that produce 100% FE of CO (*n_[i]_* = 2; *k_[i]_* = 1) or 100% FE of ethylene (*n_[i]_* = 6; *k_[i]_* = 1) are 50% or 25%, respectively.^10^ Notably, HER does not contribute to *C_2_* but still generates hydroxide that can drive CO_2_ crossover. Accordingly, we identify the CO_2_RR performance of the electrolyzers operating in neutral electrolyte that show state-of-art SPU in the references and summarized them in Supplementary Table 1. None of the reported electrolyzers operating in neutral electrolyte can achieve an SPU exceeding 30% for C_2+_ production and 44% for CO production. The theoretical SPU limitations (100% selectivity to a specific product) are listed below.

| **Product** | **CO** | **Formate** | **Acetate** | **C_2_H_4_** | **C_2_H_5_OH** | **CH_4_** | **C_3_H_7_OH** |
| --- | --- | --- | --- | --- | --- | --- | --- |
| **n** | 2 | 2 | 4 | 6 | 6 | 8 | 6 |
| **k** | 1 | 0.5 | 0.875 | 1 | 1 | 1 | 1 |
| **SPU_limit_ (%)** | 50 | 66 | 36 | 25 | 25 | 20 | 25 |

**Supplementary Table 1** Summary of the CO_2_ single-pass utilization (SPU) of the flow cells (FC) and membrane electrode assembly (MEA) in the literature using alkaline or neutral electrolytes and operating at the current densities over 100 mA cm^-2^. The stationary catholyte MEA (SC-BPMEA) operating under different conditions are listed for comparison. The upper limit SPU is simulated by substituting the FE distribution into Equation (6). The experimental SPU is calculated from Equation (3). The electrolyzers producing C_2+_ are indicated by references are blue.

| CO_2_ flow (sccm cm^-2^) | FE distribution | | | | | | | | SPU  _Upper Limit_ | SPU _Experimental_ | Experimental  : Upper Limit SPU | Cell type/ references |
| --- | --- | --- | --- | --- | --- | --- | --- | --- | --- | --- | --- | --- |
|  | H_2_ | CO | C_2_H_4_ | CH_4_ | EtOH | Acetate | Formate | Propanol |  |  |  |  |
| 3.5 | 0.07 | 0.2 | 0.45 | 0 | 0.175 | 0 | 0 | 0 | ─ | 24% | ─ | Alkaline FC^14^ |
| 50 | 0.07 | 0.06 | 0.7 | 0.02 | 0.1 | 0.05 | 0 | 0 | ─ | 0.5% | ─ | Alkaline FC^7^ |
| 8.5 | 0.36 | 0.64 | 0 | 0 | 0 | 0 | 0 | 0 | ─ | 21% | ─ | Alkaline FC^15^ |
| 25 | 0.02 | 0.95 | 0 | 0 | 0 | 0 | 0 | 0 | ─ | 7% | ─ | Neutral MEA^16^ |
| 0.4 | 0.59 | 0.00 | 0.24 | 0.08 | 0.09 | 0.025 | 0.015 | 0.01 | ─ | 30% | ─ | Neutral MEA^17^ |
| 0.6 | 0.23 | 0.77 | 0 | 0 | 0 | 0 | 0 | 0 | ─ | 44% | ─ | Neutral MEA^18^ |
| 12.5 | 0.28 | 0.72 | 0 | 0 | 0 | 0 | 0 | 0 | ─ | 40% | ─ | Alkaline MEA^19^ |
| 22.5 | 0.05 | 0.15 | 0.45 | 0.02 | 0.2 | 0.04 | 0.02 | 0.04 | ─ | 4% | ─ | Neutral FC^11^ |
| 22.5 | 0.08 | 0.18 | 0.42 | 0.02 | 0.15 | 0.03 | 0.03 | 0.05 | ─ | 3% | ─ | Neutral FC (BPM)^13^ |
| 2.34 | 0.233 | 0.098 | 0.375 | <0.001 | 0.043 | 0.015 | 0.033 | 0.024 | 27.1 ± 1.0% (22.6%) ^a^ | 18.4% | 0.7 | Neutral  SC-BPMEA (this work) |
| 1.17 | 0.231 | 0.079 | 0.402 | <0.001 | 0.049 | 0.014 | 0.031 | 0.025 | 26.2 ± 1.0% (22.6%) ^a^ | 35.5% | 1.4 | Neutral  SC-BPMEA (this work) |
| 0.58 | 0.314 | 0.054 | 0.363 | 0.001 | 0.075 | 0.012 | 0.022 | 0.035 | 22.9 ± 0.8% (19.5%) ^a^ | 61.7% | 2.7 | Neutral  SC-BPMEA (this work) |
| 0.29 | 0.641 | 0.023 | 0.223 | 0.002 | 0.067 | 0.015 | 0.014 | 0.033 | 13.3% | 78.5% | 5.9 | Neutral  SC-BPMEA (this work) |
| ^a^ SPU measurement missed 12-18% of the product FE (except the 0.29 sccm cm^-2^ case), likely because some liquid product was trapped in the stationary catholyte layer or migrated and oxidized on the anode. Therefore, the SPU reported in this work are the minimum values. The upper limit SPU values without considering the missing FE are indicated in the brackets.  Nevertheless, we take the missing FE into account for calculating the upper limit SPU of our electrolyzer to make a conservative comparison of SPU. The missing FE can be ascribed to three groups of liquid products, i.e., formate, acetate, and ethanol/propanol. However, ascribing the missing FE to formate will result in a total CO_2_ consumption exceeding the inlet CO_2_ amount; we ascribe here the missing FE to acetate, ethanol, and propanol. This simulation gives the ranges of upper limit SPU for our SC-BPMEA under different conditions. | | | | | | | | | | | | |

**SI2 Analysis of BPM** **functions**

*BPM voltage losses*

With the application of an appropriate external potential, water dissociation: H_2_O → H^+^ + OH^-^, occurs at the interface of the CEL/AEL, and the protons and hydroxides serve as charge carriers in CEL and AEL, respectively. Under standard conditions (25 ^o^C, 1 atm, with activities of H^+^ and OH^-^ at 1 M in the CEL and AEL, respectively), the electric potential across the BPM is ~0.83 V at equilibrium. The electric potential energy difference for H^+^ and OH^-^ across the BPM exactly compensates for the difference in activity (concentration) such that the electrochemical potential is the same everywhere at equilibrium.^20^ In order for net current to flow, an additional electric potential must be applied across the membrane, causing a deviation from the open-circuit value of ~0.83 V. This deviation is typically called the *water dissociation overpotential* and represents the losses associated with generating H^+^ and OH^-^ and transporting it out of the interfacial layer between CEL and AEL and out of the BPM. Often, it is stated that a BPM induces a “thermodynamic” voltage loss of 0.83 V – however, as discussed above, this is incorrect – the losses can in fact be quite small. For example, with appropriate materials and operating conditions, the cell voltage of a BPM-based water electrolyzer can in fact be lower than that of an AEM-based electrolyzer at the current density up to 500 mA cm^-2^.^20^ BPM electrolyzers can begin to split water with a total voltage of < 1.7 V, which would be impossible if there were an intrinsic 0.83 V penalty for using the BPM.

*Comparison between custom and Fumasep BPMs in SC-BPMEA*

We first conducted water-splitting measurements to compare the resistance of custom BPM and Fumasep. Supplementary Fig. 1 shows that the BPM with a simple TiO_2_ water dissociation catalyst (black plots) has lower resistance than the one without water dissociation catalyst (blue plots) and commercially available Fumasep BPM.

When being used in the SC-BPMEA with neutral anolyte (Supplementary Fig. 2), Fumasep shows similar product distributions and a slightly better CO_2_ crossover inhibiting capability (Supplementary Fig. 3), but much higher cell voltage (Supplementary Fig. 2) compared to the one based on custom BPM, which is expectable. Therefore, we adopted the custom BPM in the neutral anolyte studies.


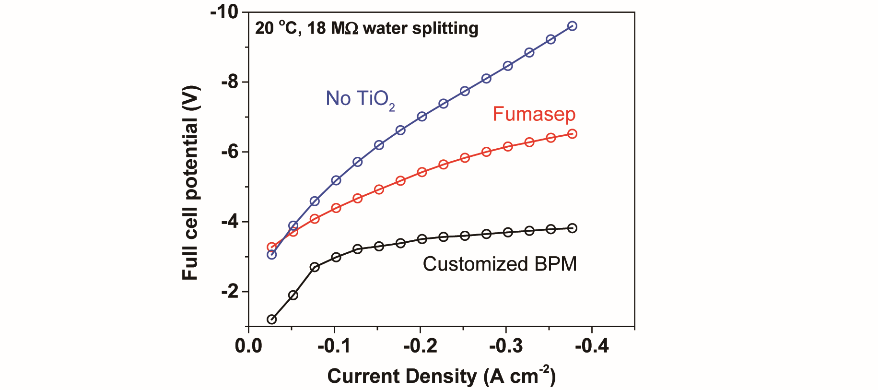


**Supplementary Fig. 1 |** The custom BPM’s water splitting performance (a layer of TiO_2_ nanoparticles as water dissociation catalyst sandwiched by CEM and AEM), commercially available BPM (Fumasep), and a membrane with CEM and AEM simply compressed together. A 5 cm^2^ Pt/C loaded hydrophilic carbon paper and a 5 cm^2^ IrO_2_ loaded Ti felt were used as the cathode and anode, respectively.





**Supplementary Fig. 2 |** The full cell voltages for the SC-BPMEA based on commercially available BPM (Fumasep), 0.1 M KHCO_3_ anolyte and the catholyte layer thicknesses of 65 μm (0.5 M K_2_SO_4_). The measurement was performed at 35 ^o^C. The FE distributions are close (< 5% deviation) to the ones using custom BPM.


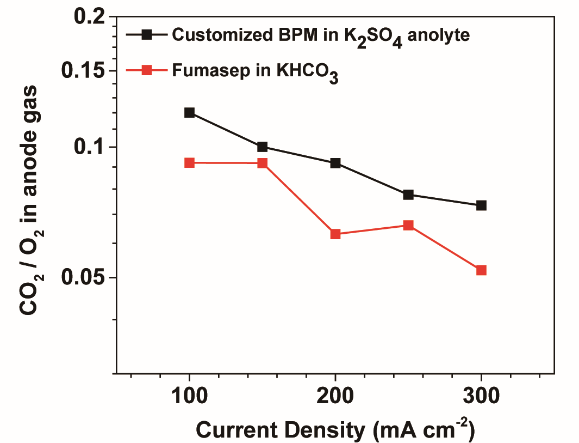


**Supplementary Fig. 3 |** The CO_2_:O_2_ ratio in anode gas phase for the SC-BPMEA using custom BPM and 0.1 M K_2_SO_4_ anolyte (black) and using commercially available BPM (Fumasep) and 0.1 M KHCO_3_ (red). The K^+^ concentrations are 1 M in the stationary catholyte layer in both cases. All the gas samples were recorded upon completion of 1 h operation at each current density. In K_2_SO_4_, the CO_2_: O_2_ ratio is close to that in KHCO_3_ for custom BPM, indicating the detected anode CO_2_ flow in the custom BPM system (Fig. 2c in the main text) was not ascribed to the acidification of bicarbonate. In an SC-BPMEA using KHCO_3_ anolyte, the CO_2_ crossover through Fumasep is similar to the case through custom BPM.

**SI3 Investigating the feasibility of the existing BPM-based MEAs for high carbon utilization in CO_2_-to-C_2+_**

The BPM-based flow cells, with both buffering and non-buffering catholyte,^11,21^ also suffer from significant CO_2_ loss and show no advantage in SPU compared with ordinary AEM-based flow cells. The flow cells usually use >1 millimetre-thick catholyte, far from the sub-150 μm-scale for efficient mass transfer of revert CO_2_. Fabricating and operating sub-150 μm-scale flowing catholyte is challenging as well. Additionally, the reverted CO_2_ is removed by the flowing catholyte and has little chance to diffuse back to the cathode for CO_2_RR. Therefore, the BPM-based flow cells are excluded from the comparison in this section.

Previous works have reported three strategies to improve the CO_2_-to-CO selectivity in BPM-based MEA electrolyzers, namely weakening the acidity of the CEL,^22^ adopting a high concentration of anolyte^23^ and inserting a >600-μm-thick buffer layer between CEL and cathode.^24^ However, we discovered that none of these systems can enable high SPU in CO_2_-to-C_2+_ conversion because of the improper comprehensive balance between cathodic local pH and the mass transfer efficiency of reverted CO_2_.


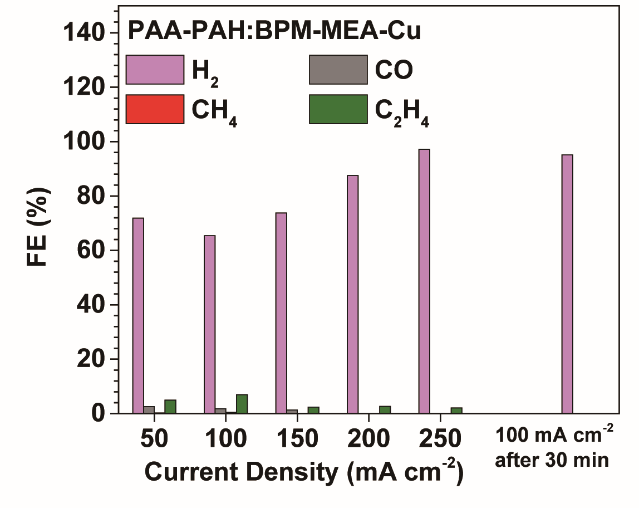


**Supplementary Fig. 4 |** The dependence of gas product FE on the current density for the BPM-based MEA referencing the system reported by Yan et al.^22^ The cathode and anode were the same Cu-based gas-diffusion electrode and IrO_2_ coated Ti felt used in this study. The membrane is a custom BPM with the CEL modified by 10 poly(acrylic acid) (PAA), and; poly(allylamine hydrochloride) (PAH) bilayers, prepared via the same procedure reported in the literature to lower the acidity.^22^ The anolyte was 1 M KHCO_3_.

Supplementary Fig. 4 shows that the PAA-PAH bilayer modified BPM can slightly improve the ethylene FE of the Cu catalyst to ca. 7% at a current density of 100 mA cm^-2^ (without modification, CO_2_RR selectivity was <0.1%). However, after 30 min of operation, the hydrogen FE increased to 95%, and the FEs for all the CO_2_R products decreased to <0.1%. We ascribe the low CO_2_R FE of this system to the fact that the CO_2_-to-C_2+_ electroproduction requires significantly higher local pH than the CO_2_-to-CO reaction on Ag. Despite the acidity being reduced by the surface modification, pH was apparently not sufficiently high to promote CO_2_RR. The lower stability of the Cu catalyst than that of Ag is also a major challenge in the BPM-based systems since the protons approaching the cathode can deteriorate Cu.


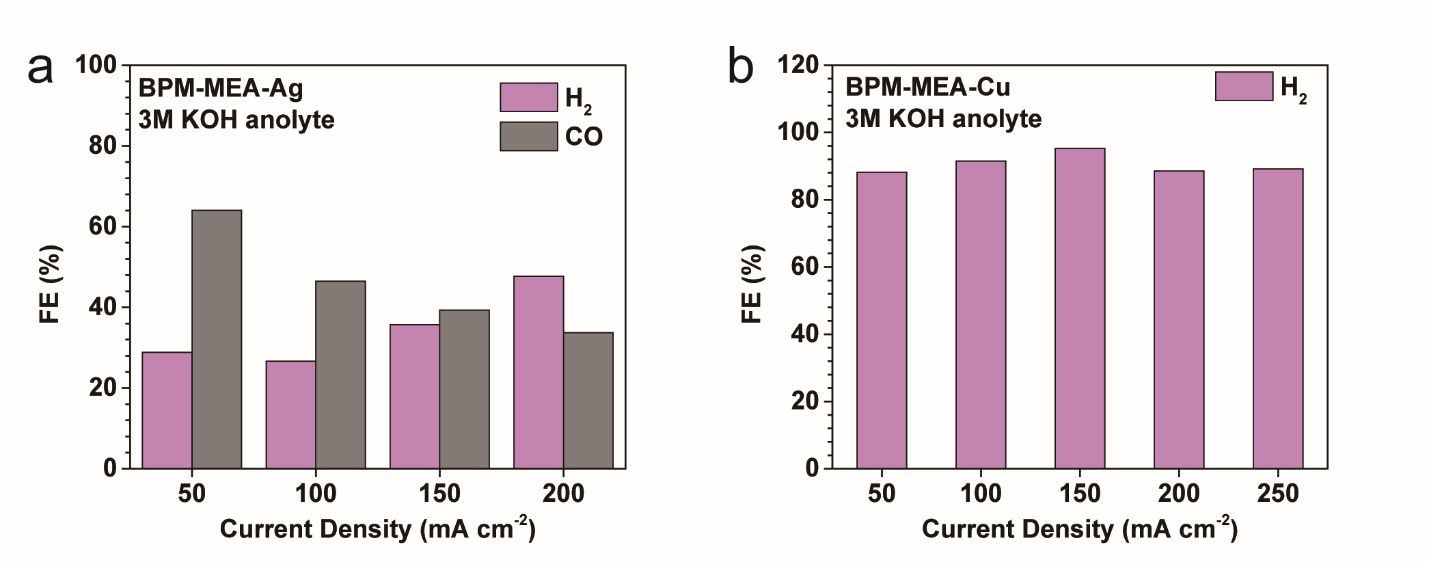


**Supplementary Fig. 5 |** The dependence of gas product FE on the current density for the BPM-based MEA referencing the system reported by Yang et al.^23^ The anolyte in both cases was 3 M KOH. The membrane was a Fumasep BPM. The anodes were IrO_2_ coated Ti felt. The cathodes were: (a) PTFE gas-diffusion layer sputtered coating with 200 nm Ag. (b) The catalyst used was the Cu-based gas-diffusion electrode similar to that used in this study.

Supplementary Fig. 5 represents the recently reported BPM-based MEA electrolyzer that uses a high-concentration anolyte to promote the selectivity of CO_2_-to-CO on an Ag electrode.^23^ This strategy lies in the crossover of the cations from anode to cathode, inducing cation effects. We reproduced this strategy on an Ag electrode, and the results (Supplementary Fig. 5a) are in good agreement with the reference.^23^ However, adopting a Cu cathode in this system, we observed ca. 90% FE for hydrogen, and all the CO_2_RR products were below detection limitation (<0.1% FE, Supplementary Fig. 5b). *This new result implies that the cathode local environment is unsuitable for C_2+_ production.*


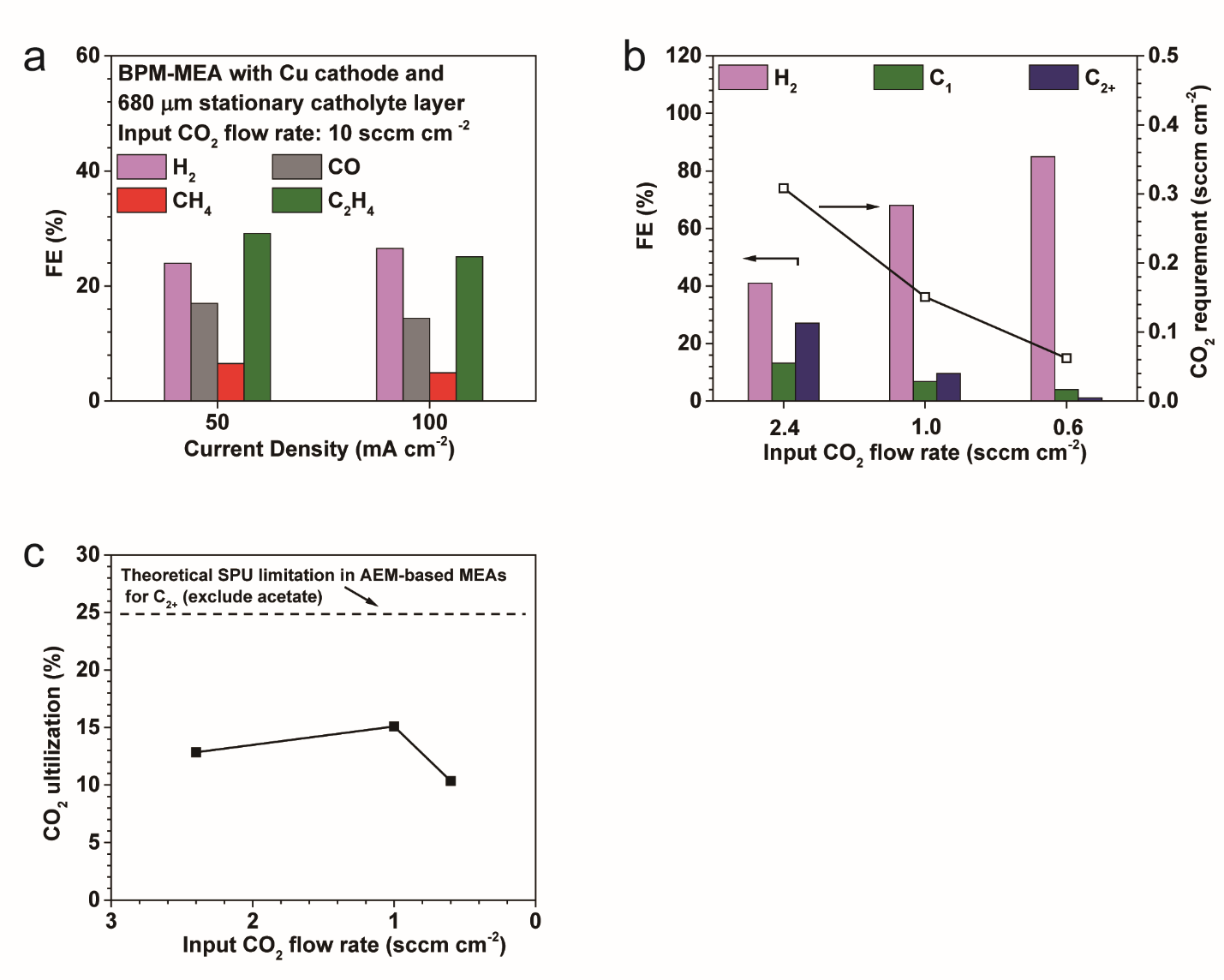


**Supplementary Fig. 6 |** The investigation on the BPM-based MEA referencing the system reported by Salvatore et al.^24^ The anolyte was 1 M NaOH. The membrane was a Fumasep BPM. The cathode and anode were Cu-based gas-diffusion electrode and IrO_2_ coated Ti felt, similar to those used in this study. Between BPM and cathode, a glass microfiber prefilter (Whatman Grade GF/D, thickness 680 μm, similar to that used in the reference^23^) saturated with 1 M NaHCO_3_. (a) The dependence of gas product FE on the current density without CO_2_ mass transport limitation (input flow rate: 10 sccm cm^-2^). Notably, the full-cell voltages were 5.4 V and 9.7 V at the current densities of 50 and 100 mA cm^-2^, respectively. (b) The dependence of the product FE and CO_2_ requirements on the input CO_2_ flow rates. The operating current density was 50 mA cm^-2^. C_1_ refers to CO, formate and methane. C_2+_ refers to ethylene, ethanol, acetate and n-propanol. (c) The total CO_2_ SPU with various input flow rates of CO_2_.

Previous reports^24,25^ proposed inserting a >600 μm-thick solid porous support layer (a buffer layer) saturated with 1 M KHCO_3_ as the stationary catholyte between the BPM and an Ag catalyst layer in an MEA. This configuration was shown to improve the selectivity of CO_2_-to-CO and suppress that of HER. With a high CO_2_ input flow rate of 10 sccm cm^-2^ (normalized by electrode area), we observed that this configuration can also promote the CO_2_R selectivity at the current density between 50 and 100 mA cm^-2^, as demonstrated in Supplementary Fig. 6a. We then explored its capability to achieve high CO_2_ SPU by gradually restricting the input CO_2_ flow rate.^6^ Shown in Supplementary Fig. 6b, as the input CO_2_ flow rate decreases, the CO_2_RR selectivity of this electrolyzer dramatically decreases along with the significant decay of the CO_2_ consumption. Consequently, the SPU of this electrolyzer reaches its maximum of ca. 15% at the input CO_2_ flow rate of 1.0 sccm cm^-2^ (Supplementary Fig. 6c). The SPU of such an electrolyzer is unable to surpass the upper limit in CO_2_-to-C_2+_ conversion. In the ‘Numerical simulation of the SC-layer’ and ‘Assessment of SPU in SC-BPMEA’ sections of the text, we discuss quantitative simulations and experimental results that reveal the underlining working mechanism of such a system. We conclude that the sluggish mass transfer of the regenerated CO_2_ inside the catholyte layer, induced by the overly thick catholyte layer, is responsible for the low CO_2_RR selectivity under restricted CO_2_ flow rates. Based on the understanding gained about the mechanism, *we illustrate a new design principle of such SC-BPMEA electrolyzers to enable high SPU for CO_2_-to-C_2+_ conversion****.***

A previous report^6^ also suggested that a DI-water layer can improve the CO_2_-to-CO selectivity for Ag. We also adopted DI-water for Cu catalyst. H_2_ dominated the cathodic products, with a FE of >94% in the current density in between 50 to 250 mA cm^-2^.

**SI4 Additional numerical simulation results on non-buffer stationary catholyte layer.**


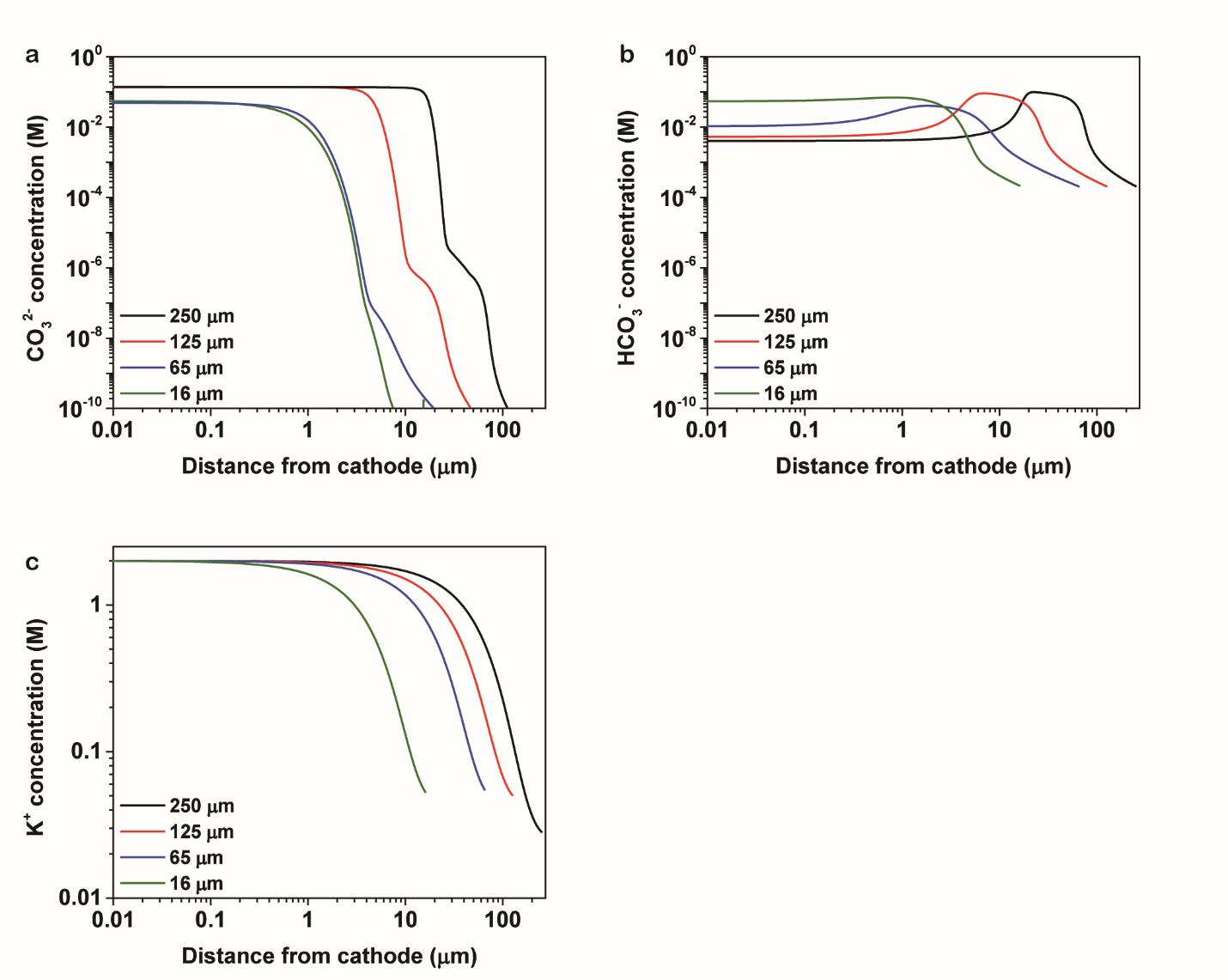


**Supplementary Fig. 7 |** Finite-element numerical simulation results of the stationary catholyte layers with 0.5 M K_2_SO_4_ and thickness from 250 to 16 μm. (a) The carbonate concentration profile. (b) The bicarbonate concentration profile. (c) The K^+^ concentration profile.

**SI5 The use of buffer catholyte (1 M KHCO_3_) in the stationary catholyte layer.**

All the previous MEAs^24,25^ inserting a stationary catholyte layer between acidic membrane and cathode used 1 M KHCO_3_ (or DI-water) as the catholyte. These devices used Ag as the catalyst, and their stationary catholyte layers were 670 to 800 μm-thick. This work focuses on C_2+_ production using Cu catalysts. It needs higher local cathode pH and more-efficient CO_2_ mass transfer to promote C-C coupling. Notably, Ag has ca. 20% CO_2_RR FE even if directly contacts BPM,^24^ while Cu gives 0%. *The previously adopted thickness and catholyte are found here unsuitable for Cu towards C_2+_ production.* The thickness impacts are extensively discussed in the main text. This section confirms that the buffer catholyte, i.e., 1 M KHCO_3_, leads to a higher energy intensity for ethylene production in SC-BPMEA.


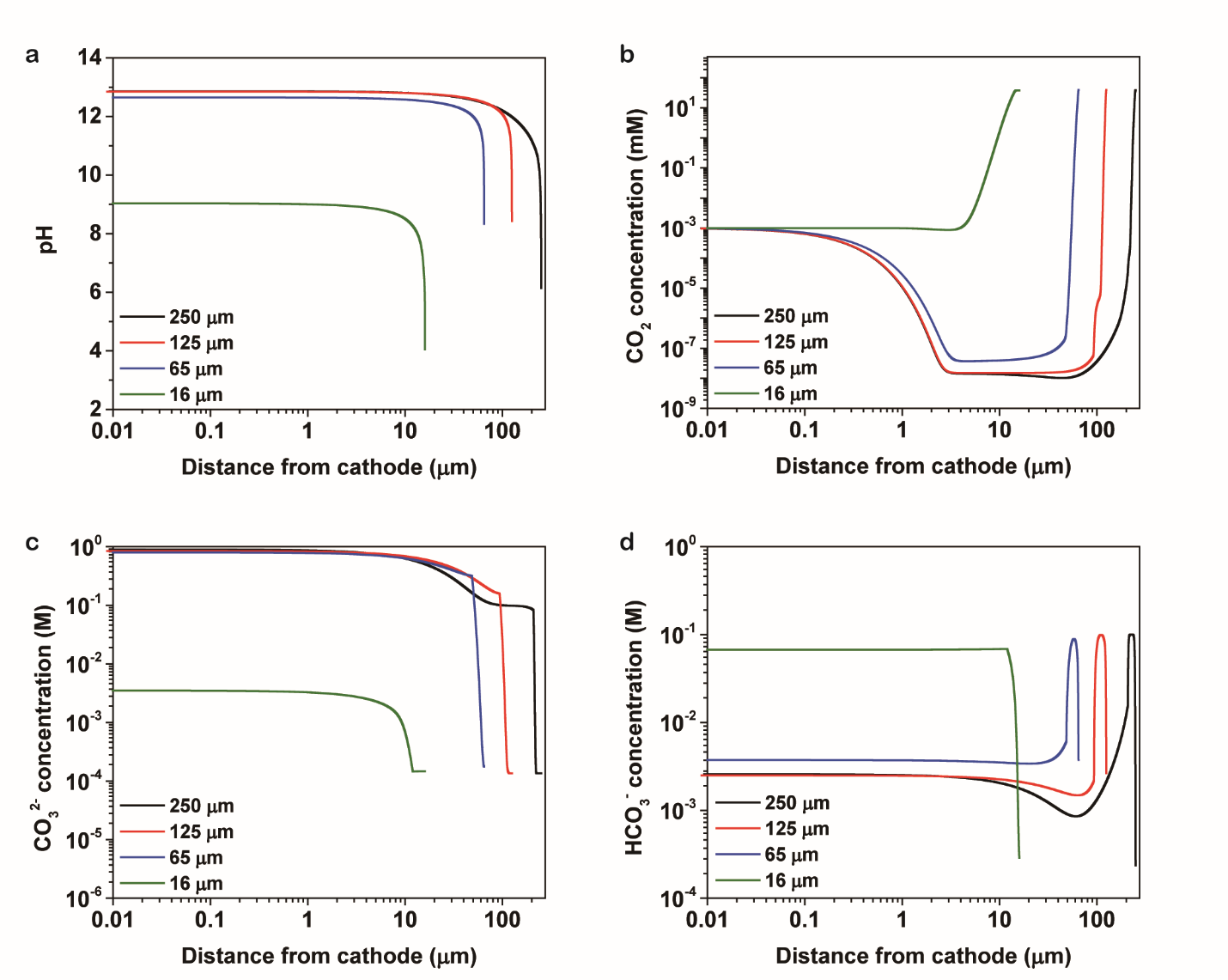


**Supplementary Fig. 8 |** Finite-element numerical simulation results of the stationary catholyte layers with 1 M KHCO_3_ and thickness from 250 to 16 μm. (a) The pH profiles. (b) The dissolved CO_2_ (aq.) concentration profiles. (c) The carbonate concentration profiles. (d) The bicarbonate concentration profiles.


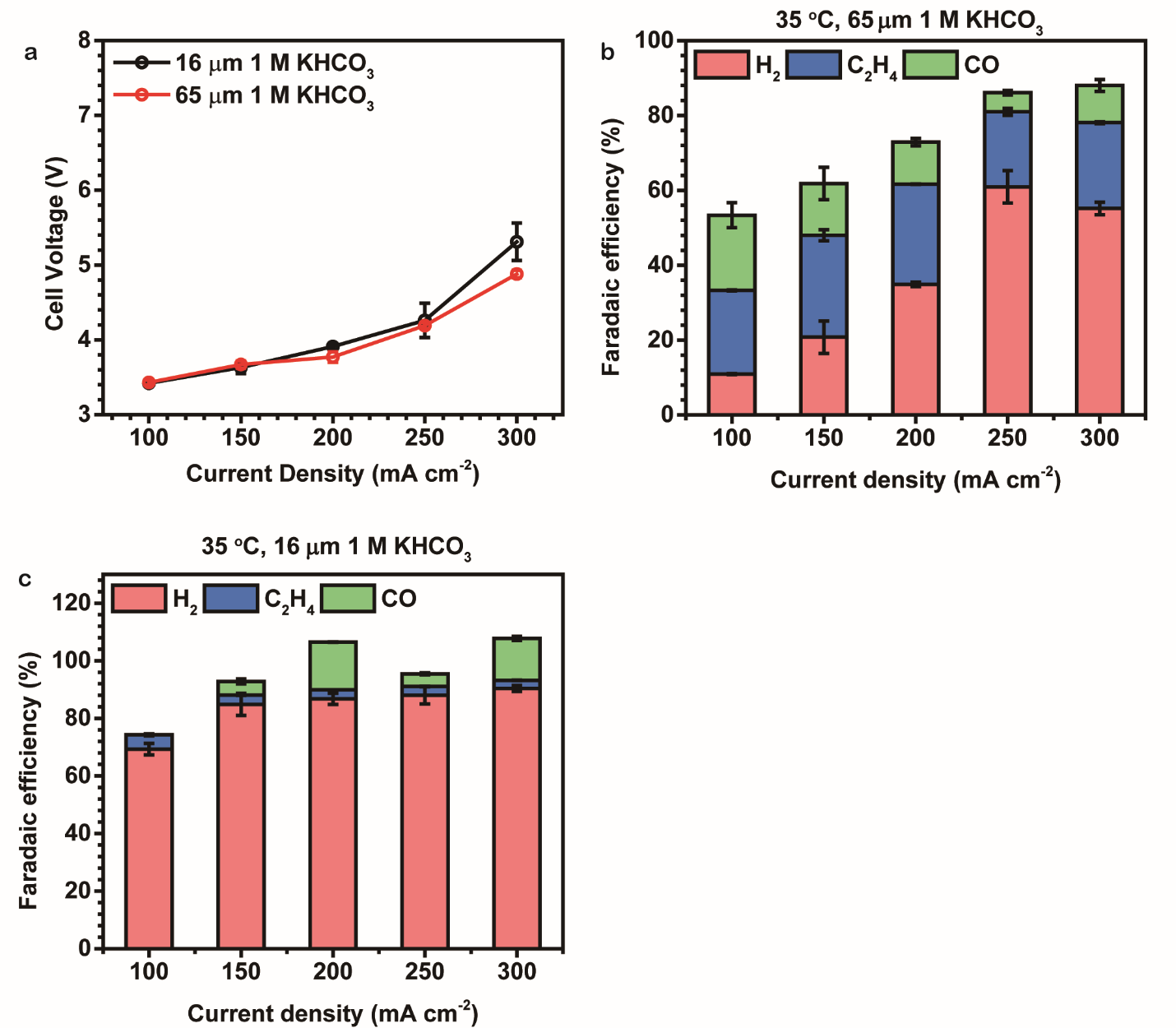


**Supplementary Fig. 9 |** The CO_2_RR performance of SC-BPMEA with 1 M KHCO_3_ as the catholyte. (a) The dependence of cell voltage with different catholyte thicknesses. (B-C) The dependence of gas FE on the current density for the catholyte thicknesses of 65 (b) and 16 μm (c). In all measurements, the temperature is 35 ^o^C, the anolyte is 0.1 M KHCO_3_ (pH = 8.2), and the CO_2_ inlet flow rate is 10 sccm cm^-2^ (normalized by electrode area).


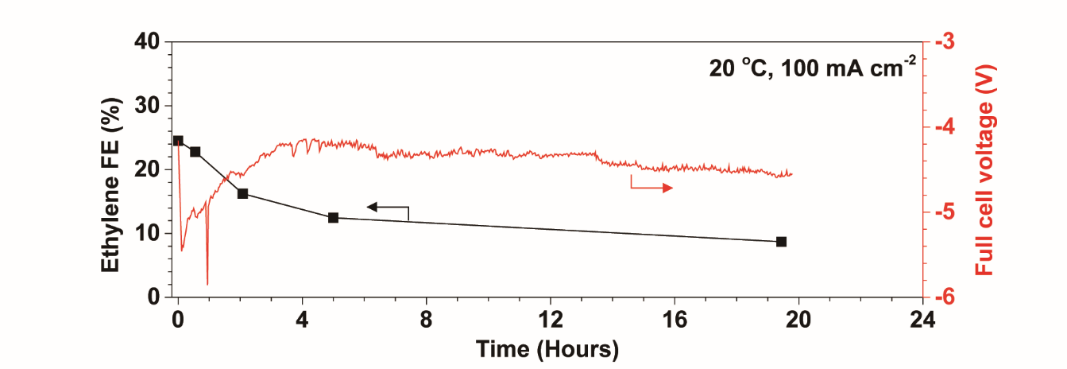


**Supplementary Fig. 10 |** Ethylene FE and cell voltage during extended operation in the SC-BPMEA with 1 M KHCO_3_ in the stationary catholyte layer and 1 M KHCO_3_ as the anolyte (similar to the previous work^24^). The measurement was carried out at 20 ^o^C with a CO_2_ flow rate of 3 sccm cm^-2^ and a current density of 100 mA cm^-2^.

The COMSOL simulations suggest that the pH gradients (Supplementary Fig. 8a) from cathode surface through the stationary catholyte layer in 1 M KHCO_3_ (ca. 4 pH units for 250, 125 and 65 μm, 6 pH units for 16 μm) are lower than those in 0.5 M K_2_SO_4_ (ca. 9 pH units for 250, 125 and 65 μm and 6 pH units for 16 μm). As confirmed experimentally, the smaller pH gradient offers a slightly lower cell voltage (Supplementary Fig. 9a). Like in 0.5 M K_2_SO_4_, the 16 μm-thick 1 M KHCO_3_ results in a low local pH near the cathode surface, and consequently, low CO_2_RR selectivity (Supplementary Fig. 9c).

Due to the buffer capacity of 1 M KHCO_3_, the virtual boundaries where carbonate/bicarbonate are regenerated to CO_2_ are right on the surface of the BPM’s CEL (Supplementary Fig. 8b). This effect causes stability issues in thicker (125 and 250 μm) stationary catholyte layer SC-BPMEAs due to CO_2_ bubble evolution. We found that using 125-μm 1 M KHCO_3_, the SC-BPMEA shows a respectable C_2_H_4_ FE of ca. 23% at the current density of 100 mA cm^-2^. However, its stability is poor in that the FE gradually decreases by > 50% after the initial 5 h. This experiment was repeated three times, and typical FEs and a cell voltage vs. time curve are displayed in Supplementary Fig. 10. With the thicker CO_2_ diffusion layer, the regenerated CO_2_ may gradually accumulate at the boundary (Scheme S2) because the concentration-gradient-driven CO_2_ diffusion flux is lower than the CO_2_ generation rate. When the accumulated CO_2_ (aq.) reaches the saturated concentration, it could bubble out periodically and physically damage the catalyst and/or stationary catholyte layers. This phenomenon may also be the reason for the periodical voltage fluctuation in Supplementary Fig. 10. Likewise, the 250 μm 1 M KHCO_3_ led to even poorer stability: the cell voltage kept increasing to 10 V in 30 min, with >80% H_2_ FE.

At 100 mA cm^-2^, SC-BPMEA with 65 μm 1 M KHCO_3_ delivers a lower H_2_ FE (Supplementary Fig. 9b) than SC-BPMEA with 65 μm 0.5 M K_2_SO_4_ due to the higher local pH (Supplementary Fig. 8a). However, its maximum ethylene FE (27% at 150 mA cm^-2^) is lower than that of 65 μm 0.5 M K_2_SO_4_ (41% at 200 mA cm^-2^), leading to a loss of ethylene-specific energy intensity that cannot be compensated by a ~0.15 V lower cell voltage.

To this end, a non-buffer catholyte is adopted in the main part of this study.

**SI6 Exploration of the operating conditions for SC-BPMEA with non-buffering catholyte.**

A previous report also suggested DI water can be the stationary catholyte for Ag catalyst, which showed similar performance to 1 M KHCO_3_.^24^ In the SC-BPMEA, DI-water catholyte gives a >94% FE towards hydrogen, and a high full-cell voltage of 6-9 V on Cu, as shown in Supplementary Fig. 11a. On the other hand, the cation effect^26–29^ appears to play an important role in the performance of SC-BPMEA. Under an external potential, cations such as K^+^ form an electrochemical double layer on the catalyst surface, causing changes in polarity, absorption preference, local pH, and local CO_2_ concentration, as observed and modelled before.^27,28^ Increasing K^+^ concentration from 0 to 2 M in the stationary catholyte layer enhances CO_2_RR selectivity. At optimized current densities (Supplementary Fig. 11a-c and the main text Fig. 3d), the ethylene FE increases from 0.5% (0 M) and 2.5% (0.5 M) to 25% (1 M) and 27% (2 M). The enhancement of CO_2_RR selectivity with increasing K^+^ concentration suggests an important role for cation effects in promoting CO_2_RR in SC-BPMEA. Despite a higher ethylene FE and a lower hydrogen FE, 2 M K^+^ in the stationary catholyte results in carbonate salt precipitation at the backside of the carbon paper, which causes the loss of K^+^ and obstructs the mass transport of CO_2_ over time.^30^ Therefore, we adopt 1 M K^+^ in the stationary catholyte layer. This cation effect can be extended to other cation species such as Na^+^ (Supplementary Fig. 11d) and Cs^+^ (Supplementary Fig. 11e). Under the same operation conditions, SC-BPMEA with a 65 μm 1 M Na^+^ catholyte shows similar performance to SC-BPMEA with a 65 μm 1 M K^+^ catholyte, while SC-BPMEA with Cs^+^ catholyte does not yield a high CO_2_RR selectivity (Supplementary Fig. 11f).

Previous studies advised that larger cations promote higher ethylene FE.^27,28^ However, we observed a decreasing ethylene FE with increasing cation size. We performed the CO_2_RR with the Cu-based gas-diffusion electrode in a flow cell with a 1 cm-thick stationary 0.5 M Cs_2_SO_4_ (same setup to the measurement for Supplementary Fig. 13c). We discovered that in such a thick catholyte, larger cations promote higher ethylene FE. Specifically, the maximum ethylene FE in 1 M Cs^+^ is ~50% (Supplementary Fig. 11g), higher than ~43% in 1 M K^+^ (Supplementary Fig. 13c). According to previous studies, the trend of the cation effects on the C_2+_ selectivity is greatly affected by hydrolysis of the hydrated cations.^27^ Larger cations enhance the C_2+_ selectivity when hydrolyzed as M^+^(H_2_O)_n_ + H_2_O ⇌ MOH(H_2_O)_(n-1)_ + H_3_O^+^ while the non-hydrolyzed cations (e.g. acidic pH can suppress) show opposite trend. ^27^ The simulation in the main text (Fig. 2b) displays that the bulk pH in SC-BPMEA is acidic because of the protons generated from the BPM. As such, we observe that larger cations lower C_2+_ selectivity in SC-BPMEA.

The operating temperature also affects the CO_2_RR selectivity. The Cu catalyst in this work reaches the maximum ethylene FE of 36% (among 125 μm 0.5 M K_2_SO_4_) at 35 ^o^C (Supplementary Fig. 12), consistent with previous discoveries.^17^


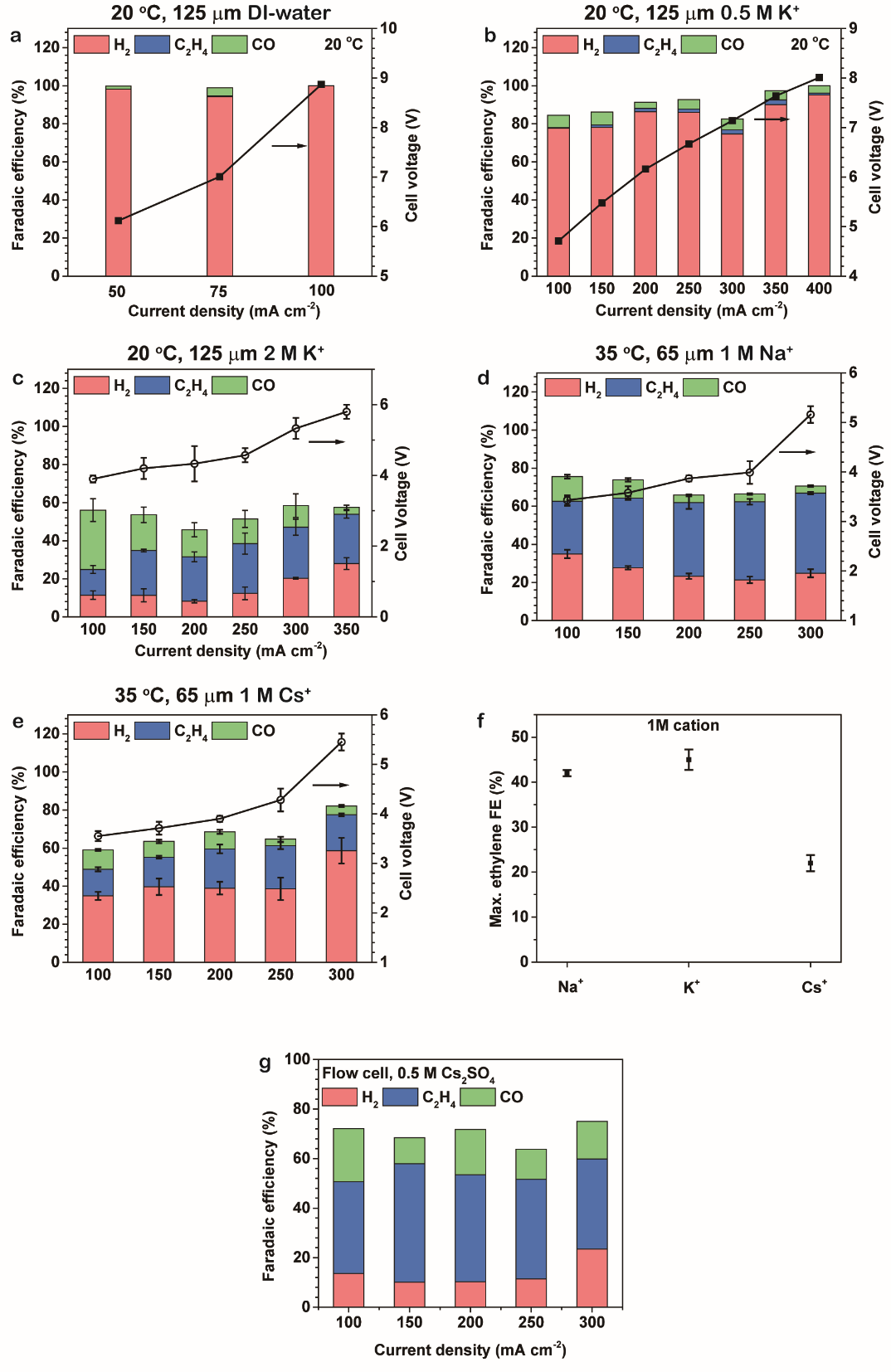


**Supplementary Fig. 11 |** Cation effects on the CO_2_RR performance of SC-BPMEA. (a) 125 μm DI-water; (b) 125 μm 0.25 M K_2_SO_4_; (c) 125 μm 2 M KCl; (d) 65 μm 0.5 M Na_2_SO_4_; (e) 65 μm 0.5 M Cs_2_SO_4_. (f) The dependence of maximum ethylene FE on cation species in a 65 μm catholyte layer. The results for 125 μm 0.5 M K_2_SO_4_ (1 M K^+^) at 20 ^o^C are shown in Supplementary Fig. 12a and 12b. The results for 65 μm 0.5 M K_2_SO_4_ (1 M K^+^) at 35 ^o^C are shown in Fig. 3e in the main text. In all cases, The CO_2_ flow rate is 10 sccm cm^-2^. (g) The CO_2_RR performance of the Cu nanoparticles in a 1 cm-thick, stationary 0.5 M Cs_2_SO_4_.


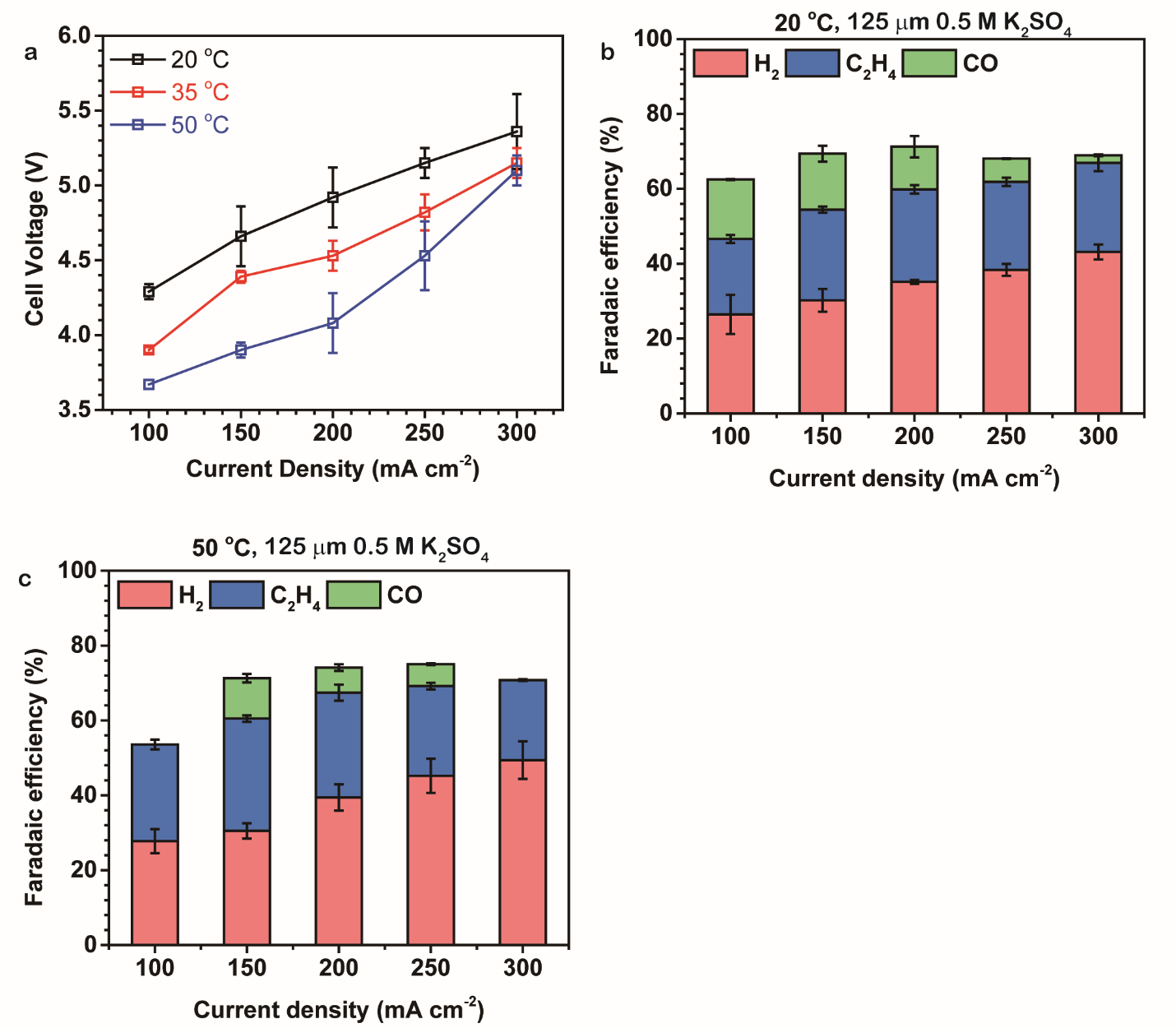


**Supplementary Fig. 12 |** Temperature effects on the CO_2_RR performance of SC-BPMEA. (a) The dependence of cell voltage on operating temperature. (b-c) The dependence of gas FE on the current density for the operating temperatures of 20 ^o^C (b) and 50 ^o^C (c). The FE for 35 ^o^C is shown in Fig. 3d in the main text. In all measurements, the SC-layer is 125 μm with 1 M K^+^. The anolyte is 0.1 M KHCO_3_ (pH = 8.20), the CO_2_ inlet flow rate is 10 sccm cm^-2^.

**SI7 Analysis on the cell voltage of SC-BPMEA operating at 35 ^o^C, 200 mA cm^-2^, with 0.1 M KHCO_3_ anolyte and 65 μm 0.5 M K_2_SO_4_ catholyte layer.**


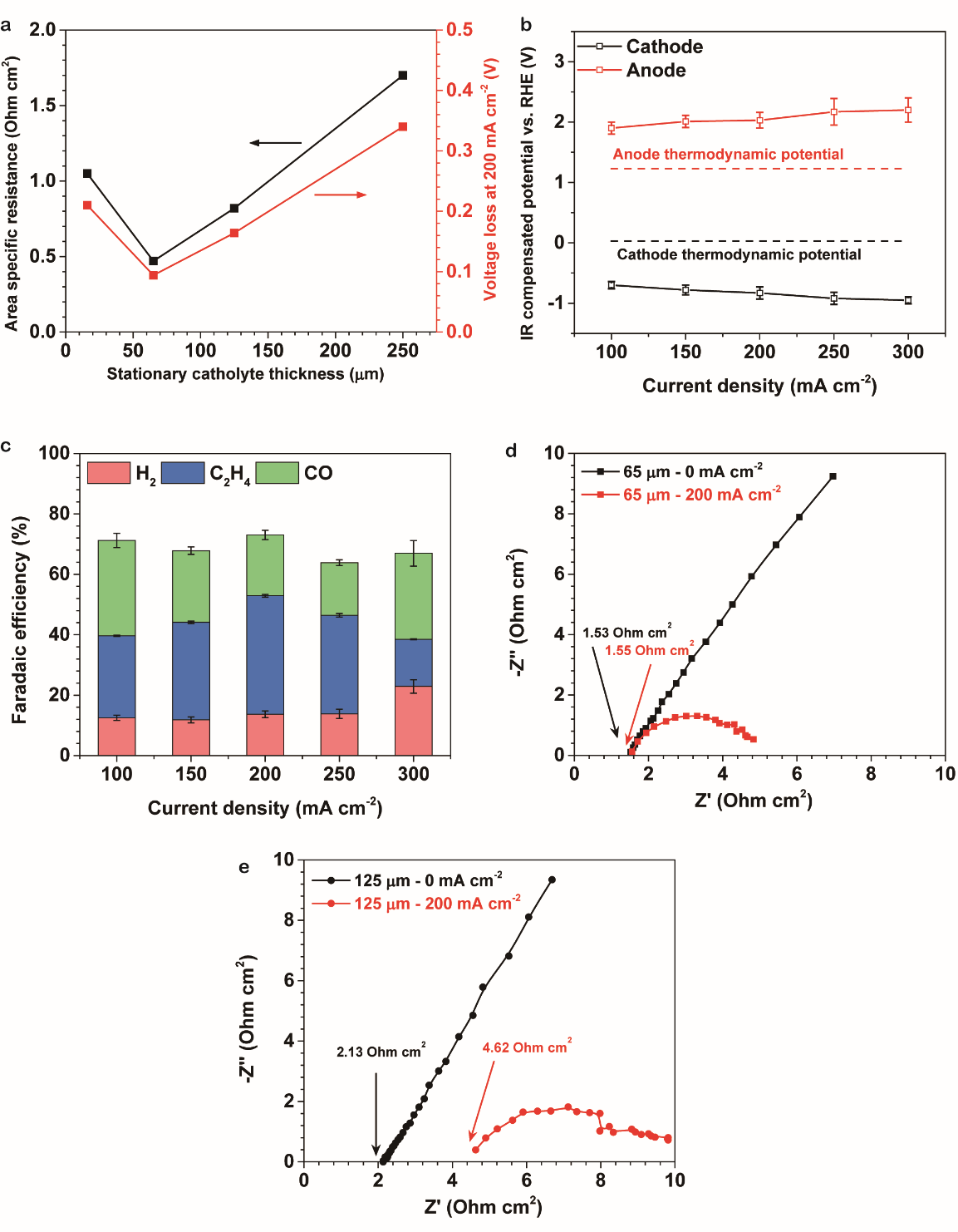


**Supplementary Fig. 13 |** Investigations on the cell voltage of the SC-BPMEA using the custom BPM. (a) The ohmic resistance (black) and the contribution to voltage loss (red) of the stationary catholyte layer with various thicknesses. The errors in these measurements are below 1%. (b) The iR-compensated voltage of cathode (black) and anode (red) at different current densities. (c) The FE distribution of the CO_2_RR gas products at different current densities. (d, e) The Nyquist plots of SC-BPMEAs with 65-μm or 125-μm SC layer, at the biased current density of 0 (black) and 200 mA cm^-2^ (red). The 200 mA cm^-2^ plots were recorded after stabilized for 20 min.

The ohmic resistance of the stationary catholyte layer (Supplementary Fig. 13a) was determined by measuring the EIS of the cell [stainless steel plate | stationary catholyte layer | stainless steel plate], which is a conventional method to measure the resistance of the electrolytes. ^31^ The voltage loss due to the stationary ohmic resistance is calculated from [area specific resistance] times [current density]. The 16-μm catholyte exhibits higher resistance than the 65- and 125-μm ones, although thinner. This likely originates from the fact that the 16 μm-thick support has a porosity of only 10%-20%, significantly lower than the 65 μm (80%), 125 μm (70%) and 250 μm (70%) ones. The resistance of the 65 μm, 125 μm and 250 μm thick catholyte layer is dominated by the ion migration path length, showing a nearly linear correlation with thickness.

The cell voltage of the SC-BPMEA is ascribed to various components: thermodynamic, cathode overpotential, anode overpotential, ohmic loss, Nernstian pH loss and other losses (interface, BPM water dissociation, etc.). Combining the experimental results and COMSOL simulation, we evaluated the cell voltage distribution as follows:

The thermodynamic voltage is 1.20 V, according to previous reports.^6,17^

The cathode overpotential was found to be 0.78 ± 0.10 V as measured in a three-chamber (catholyte, anolyte and gas) 1 cm^2^ flow-cell setup reported before.^6^ 0.5 M K_2_SO_4_ was used as the catholyte, where the catholyte was kept stationary, and anolyte was circulated. 10 sccm cm^-2^ CO_2_ was fed into the gas chamber. The cathode is the same one used in SC-BPMEA, i.e., the Cu nanoparticles coated carbon paper, and an Ag/AgCl (sat. KCl) was inserted into the catholyte as the reference. A Fumasep BPM separated catholyte (CEL face) and anolyte (AEL face) chambers. The potentials were recorded with iR compensation, in which R was determined by electrochemical impedance spectra (EIS) before the experiment. Following a previously reported approach, the Ag/AgCl reference was calibrated to a reversible hydrogen electrode (RHE). ^32^ The flow cell, catholyte, and anolyte were heated to 35 ^o^C. As shown in Supplementary Fig. 13b, the cathode overpotential is 0.78 ± 0.10 V at 200 mA cm^-2^. The FE distributions at various current densities (Supplementary Fig. 13c) in the flow cell are similar to those in SC-BPMEA (Fig. 3c in the main text), validating the feasibility of this overpotential measurement.

The anode overpotential was found to be 0.78 ± 0.13 V as measured per the same approach as cathode overpotential, except that the reference electrode was inserted into anolyte, and both cathode and anode chambers were circulated with 0.1 M KHCO_3_. This is a fairly high overpotential for the anode evolving oxygen, in part due to the neutral pH buffer used at the anode. Improved performance is certainly readily possible under locally basic anode conditions and/or with improved anode architectures.

An ohmic loss of 0.31 ± 0.01 V was determined by the product of the area-specific high-frequency resistance (HFR) of SC-BPMEA and current density. The area-specific HFR of SC-BPMEA was measured by EIS at the open-circuit voltage and 35 ^o^C. The high-frequency intercept on the real axis is used to evaluate HFR (Supplementary Fig. 13d).^33^

Nernstian pH loss was estimated to be 0.51 V the cathode-surface-to-catholyte-bulk pH gradient using the following equation:^6^

$$V_{Nernstian}=0.059\times\left( {pH}_{surface}-{pH}_{bulk} \right)$$

The sum of the above voltages is 3.58 V which is 0.24 V lower than the experimentally measured voltage of 3.82 V. This is assigned to ‘other’ voltage losses, including water dissociation overpotential,^20^ interface resistance,^34^ etc. Therefore, we conclude that the BPM’s water dissociation overpotential (likely no more than 0.24 V, consistent with previous work on these systems) in the SC-BPMEA is small compared to the other voltage losses, though further improvement of the BPM would be important particularly for CO_2_ electrolyzers operating at larger current densities, for example 1 A cm^-2^, and as the other losses are mitigated.

The EIS results (Supplementary Fig. 13d and 13e) of the SC-BPMEA agree with the prediction of the COMSOL simulation (Fig. 2a in the main text). With an SC-layer thicker than 125 μm, the generated CO_2_ bubble evolves inside the SC-layer when the current is applied, elevating the ohmic resistance of the cell voltage. As shown in Supplementary Fig. 13d, the SC-BPMEA with 65 μm SC-layer shows similar HFR values with or without the biased current (200 mA cm^-2^). Contradictory, the biased current to the SC-BPMEA with 125 μm SC-layer (Supplementary Fig. 13e) increased the HFR from 2.13 to 4.62 ohm cm^2^ due to the evolution of CO_2_ microbubbles under the current.

Without the biased current, the HFR of the SC-BPMEA with 125 μm SC-layer is 0.60 ohm cm^2^ higher than that with 65 μm SC-layer, referring to a cell voltage increment of 0.12 V at 200 mA cm^-2^. This cannot explain the 0.67 V cell voltage difference (Fig. 3a in the main text). Differently, with the applied current density of 200 mA cm^-2^, the 125 μm SC-layer increases the ohmic resistance by 3.07 ohm cm^2^, referring to a cell voltage increment of 0.61 V at 200 mA cm^-2^, close to the 0.67 cell voltage difference (Fig. 3a in the main text) observed.

**SI8 The FE distributions of the SC-BPMEA with a 16 μm 0.5 M K_2_SO_4_ SC layer.**


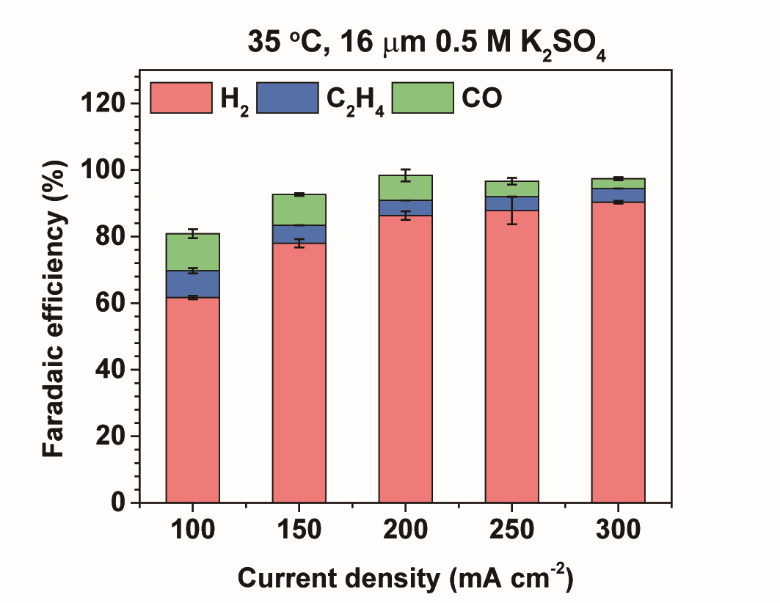


**Supplementary Fig. 14 |** The CO_2_RR performance of SC-BPMEA with 16 μm SC layer. The corresponding cell voltages are shown in Fig. 3a in the main text. The anolyte is 0.1 M KHCO_3_ (pH = 8.2) in all measurements, and the CO_2_ inlet flow rate is 10 sccm cm^-2^.

**SI9 Additional CO_2_ SPU results for SC-BPMEAs with different SC-layer thickness.**


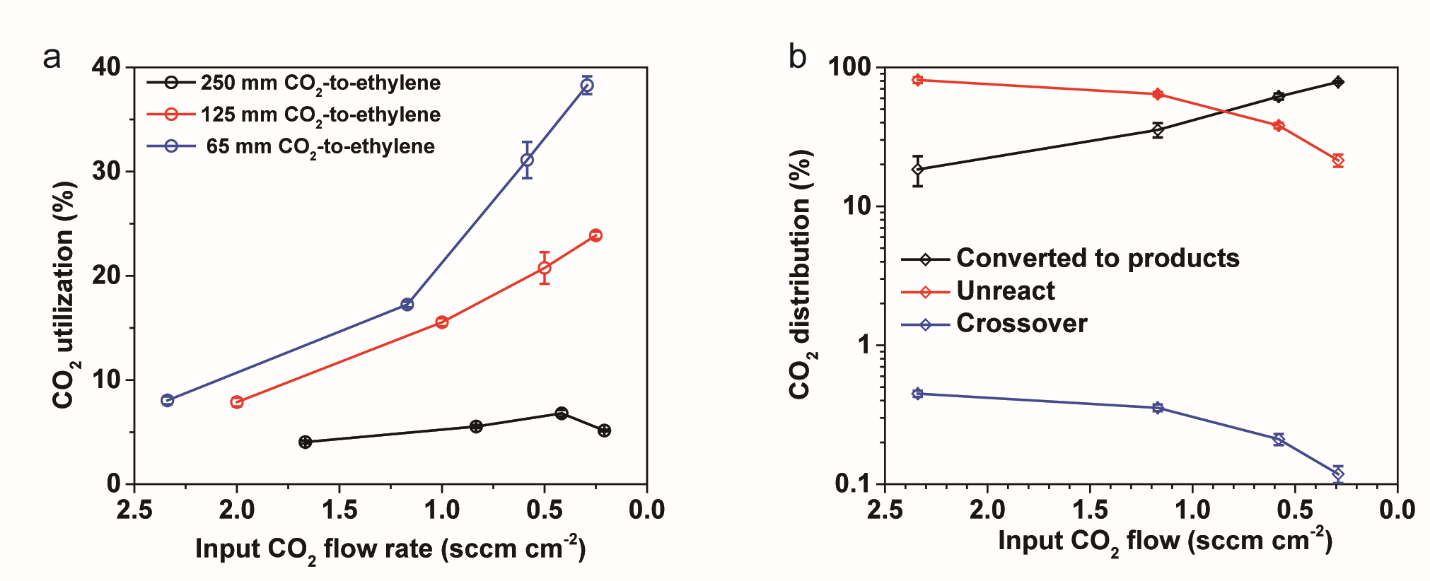


**Supplementary Fig. 15 |** (a) The CO_2_-to-ethylene conversions of the SC-BPMEAs with different thickness of 0.5 M K_2_SO_4_ SC-layers at different input CO_2_ flow rates. (b) The CO_2_ distributions of the SC-BPMEA with 65 μm-thick 0.5 M K_2_SO_4_, operating at different input CO_2_ flow rates and a current density of 200 mA cm^-2^.

**SI10 The compatibility of SC-BPMEA with acidic and basic anolytes.**

For the SC-BPMEA using acidic anolyte, the custom BPM enables a lower cell voltage than Fumasep. However, this system always fails within ~4-6 h of continuous operation due to an apparent short-circuit issue, and the typical voltage versus operation duration is shown in Supplementary Fig. 16b. We suspect this is caused by the growth of Cu dendrites that physically penetrated through BPM and contact with the anode. Cu could be partially dissolved by acid and electrochemically re-deposited onto the catalyst layer, forming sharp dendrites.^35^ Differently, we found that the Fumasep-based SC-BPMEA is more stable, probably because Fumasep is mechanically reinforced and thus more rigid, so Cu dendrites cannot easily penetrate.


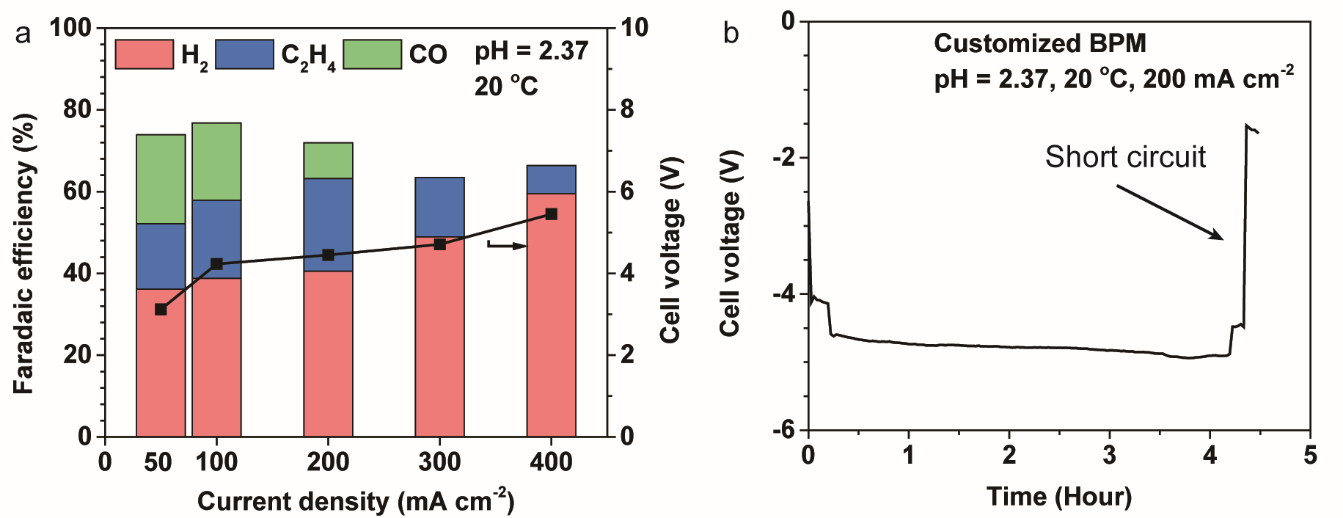


**Supplementary Fig. 16 |** (a) The FE and full cell voltage for the SC-BPMEA based on 125 μm 0.5 M K_2_SO_4_, custom BPM and 0.1 M H_3_PO_4_ + 0.5 M K_2_SO_4_ anolyte. (b) The cell voltage versus operating time diagram of the cell.


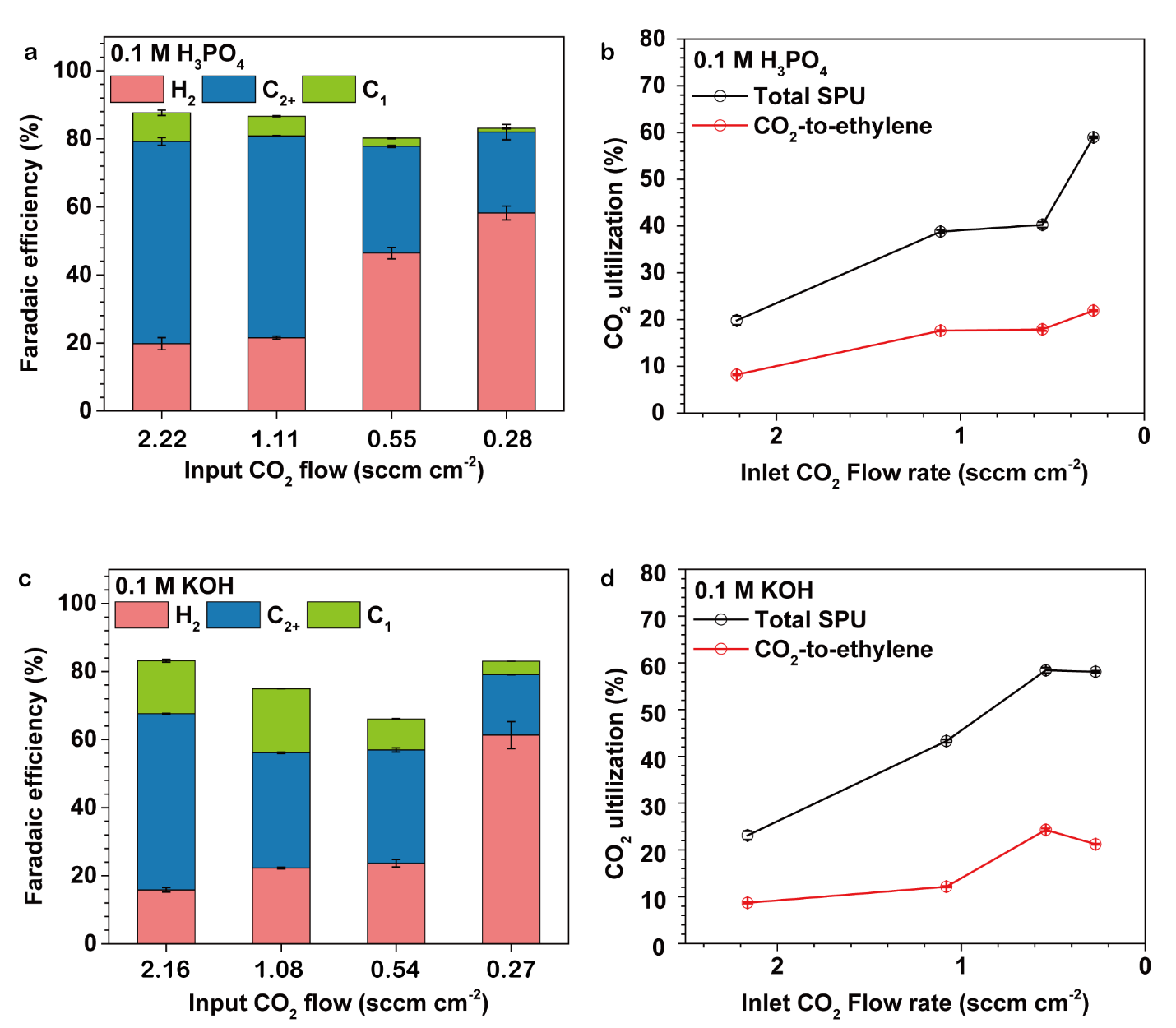


**Supplementary Fig. 17 |** Measurements of CO_2_ SPU of the SC-BPMEAs with 125 μm 0.5 M K_2_SO_4_, operating at 300 mA cm^-2^ (a, b) or 200 mA cm^-2^ (c, d). The operating anolytes are (a, b) 0.1 M H_3_PO_4_ + 0.5 M K_2_SO_4_ (pH = 2.3) and (c, d) 0.1 M KOH (pH = 13.3). All the experiments were conducted at 35 ^o^C. (a, c) The FE distributions at different input CO_2_ flow rates. (b, d) The total CO_2_ SPU and CO_2_-to-ethylene conversion at different input flow rates.

**SI11 Stability of SC-BPMEA with 65 μm 0.5 M K_2_SO_4_ and restricted CO_2_ flow rate**


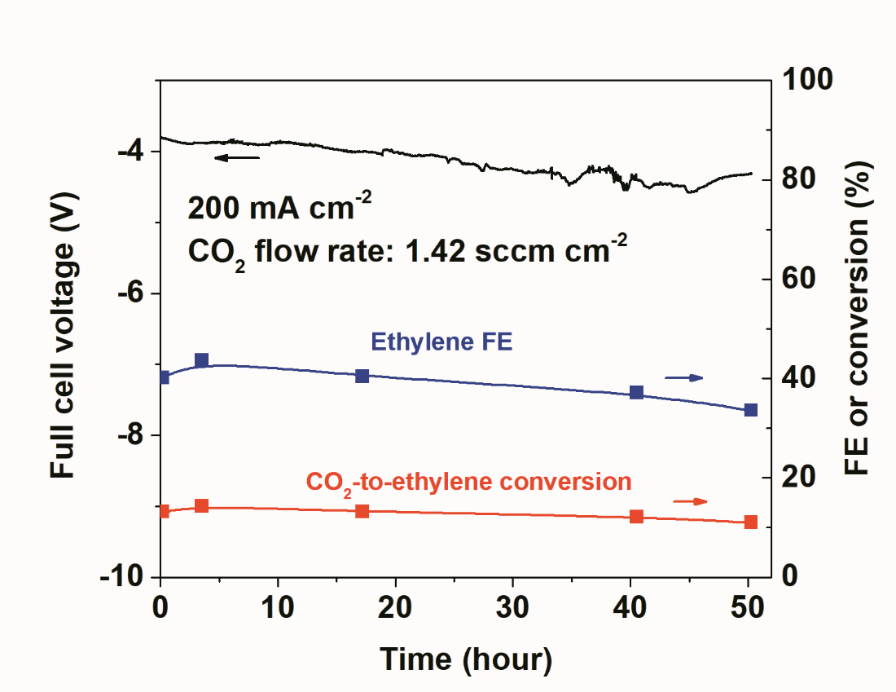


**Supplementary Fig. 18 |** The extended operation of the SC-BPMEA with 65 μm 0.5 M K_2_SO_4_ catholyte, operating at 35 ^o^C at a current density of 200 mA cm^-2^ and a CO_2_ flow rate of 1.42 sccm cm^-2^. Notably, all the previous neutral-media electrolyzers measured their stability with unrestricted CO_2_ supply (CO_2_ flow rates > 10 sccm cm^-2^), and the CO_2_-to-ethylene conversions were below 1.5%. After operating with 52 h, the ethylene FE dropped to ca. 32%. To diagnose the degradation mode, we dissembled the SC-BPMEA, washed and dried the catalyst and re-assembled it with fresh BPM, anode, electrolyte and SC-layer. The device showed an ethylene FE of 34% and a cell voltage of 4.05 V. This result implies that the degradation of the cathode is likely the main reason of the ethylene FE decrease after 52 h of operation.

**SI12 Additional energy assessments of the alkaline electrolyzer and SC-BPMEA operating at different CO_2_ input flow rates.**

In alkaline electrolyzer, CO_2_ and OH^-^ react to form carbonate continuously. This carbonate has to be recovered to maintain the CO_2_RR performance of such a system, consuming 1500 kWh (5.5 GJ) per tonne CO_2_^12^. In the alkaline CO_2_RR electrolyzers, ca. 63 tonne of CO_2_ transforms to carbonate to produce 1 tonne of ethylene, representing an energy penalty of 97,000 kWh or 350 GJ^12^. Considering a cheapest electricity cost of 0.020 $ per kWh, such an energy penalty costs at least $1,900 per tonne of ethylene, while its market price is $800-1000 per tonne^36^. The alkaline electrolyzers thus do not allow for ethylene electrochemical production to be yet profitable.

**Supplementary Table 2** Comparison of the energy intensity between various CO_2_-to-ethylene electrolyzers and this work. All the energy costs are normalized per ton ethylene produced. The flow rate of 2.34 sccm cm^-2^ is not shown here because it results in an SPU of 18% and an ethylene FE of 37%, which requires more GJ to produce one-ton ethylene than the flow rate of 1.17 sccm cm^-2^. A flow-cell working in strong alkaline (7 M KOH)^7^ is presented for comparison.

| **Metrics** | | **This work (0.58 sccm cm^-2^)** | **This work (0.36 sccm cm^-2^)** | **Science 2018, 783** |  |
| --- | --- | --- | --- | --- | --- |
| Cell type | | SC-BPMEA | SC-BPMEA | Flow-cell |  |
| Electrolyte | | Neutral | Neutral | 7 M KOH |  |
| Full cell voltage (V) | | 3.82 | 3.82 | 2.4 |  |
| Ethylene FE (%) | | 36 | 22 | 70 |  |
| Current density (mA cm^-2^) | | 200 | 200 | 110 |  |
| Total CO_2_ SPU (%) | | 62 | 78 | 1.7 |  |
| CO_2_-to-ethylene (%) | | 31 | 38 | - |  |
|  | **Energy consumption (GJ per ton ethylene)** | | | | |
| Electrolyzer electricity | | 438 | 718 | 142 |  |
| Cathode separation | | 13 | 20 | 9 |  |
| Anode separation | | 0 | 0 | 0 |  |
| Electrolyte regeneration | | 0 | 0 | 497 |  |
| **Overall energy** | | **455** | **744** | **648** |  |

**SI13 Additional information for COMSOL one-dimensional modeling**

**Supplementary Table 3** The chemical reactions at the catholyte region and the corresponding forward *k*_f_ rate constants and reverse *k*_r_ rate constants used in simulation^37^.

| Reaction | *k*_f_ | *k*_r_ |
| --- | --- | --- |
| $CO_{2}+H_{2}O\leftrightarrow H^{+}+{HCO}_{3}^{-}$ | 0.036 [s^-1^] | 7.83×10^4^ [M^-1^s^-1^] |
| $HCO_{3}\leftrightarrow H^{+}+{CO}_{3}^{2-}$ | 2.5 [s^-1^] | 5×10^10^ [M^-1^s^-1^] |
| $CO_{2}+OH^{-}\leftrightarrow{HCO}_{3}^{-}$ | 2.23×10^3^ [M^-1^s^-1^] | 4.85×10^-5^ [M^-1^s^-1^] |
| ${HCO}_{3}^{-}+OH^{-}\leftrightarrow{CO}_{3}^{2-}+H_{2}O$ | 6×10^9^ [M^-1^s^-1^] | 1.2 [s^-1^] |
| $K_{2}SO_{4}\leftrightarrow2K^{+}+{SO}_{4}^{2-}$ | 1×10^7^ [s^-1^] | 7.96×10^7^ [M^-2^s^-1^] |
| $H_{2}O\leftrightarrow H^{+}+ OH^{-}$ | 10^8^ [s^-1^] | 10^19^ [M^-1^s^-1^] |

**Supplementary Table 4** The species diffusion coefficients used in simulation^38–41^.

| **Species** | ${\mathbf{Diffusion coefficients}\boldsymbol{D}}_{\boldsymbol{i}}\boldsymbol{(}{\boldsymbol{10}^{\boldsymbol{-9}}\boldsymbol{m}}^{\boldsymbol{2}}\boldsymbol{s}^{\boldsymbol{-1}}$**)** |
| --- | --- |
| CO_2_ | 1.91 |
| H_2_O | 2.57 |
| K_2_SO_4_ | 1.39 |
| KHCO_3_ | 1.20 |
| K^+^ | 1.98 |
| H^+^ | 9.31 |
| OH^-^ | 5.26 |
| HCO_3_^-^ | 1.185 |
| CO_3_^2-^ | 0.923 |
| SO_4_^2-^ | 1.07 |

**Supplementary Table 5** The sechenov coefficients used in simulation^42^.

| **Species** | **Sechenov coefficients** |
| --- | --- |
| h_G,0,CO2_ | -0.0172 |
| h_T,CO2_ | -0.000338 |
| h_K_ | 0.0922 |
| h_OH_ | 0.0839 |
| h_HCO3_ | 0.0967 |
| h_CO3_ | 0.1423 |

**Supplementary References**

1. García de Arquer, F.P., Dinh, C.T., Ozden, A., Wicks, J., McCallum, C., Kirmani, A.R., Nam, D.H., Gabardo, C., Seifitokaldani, A., Wang, X., et al. (2020). CO2 electrolysis to multicarbon products at activities greater than 1 A cm−2. Science *367*, 661–666.

2. Li, F., Thevenon, A., Rosas-Hernández, A., Wang, Z., Li, Y., Gabardo, C.M., Ozden, A., Dinh, C.T., Li, J., Wang, Y., et al. (2020). Molecular tuning of CO2-to-ethylene conversion. Nature *577*, 509–513.

3. Ross, M.B., De Luna, P., Li, Y., Dinh, C.T., Kim, D., Yang, P., and Sargent, E.H. (2019). Designing materials for electrochemical carbon dioxide recycling. Nat. Catal. *2*, 648–658.

4. Li, F., Li, Y.C., Wang, Z., Li, J., Nam, D.H., Lum, Y., Luo, M., Wang, X., Ozden, A., Hung, S.F., et al. (2020). Cooperative CO2-to-ethanol conversion via enriched intermediates at molecule–metal catalyst interfaces. Nat. Catal. *3*, 75–82.

5. Wang, Y., Wang, Z., Dinh, C.T., Li, J., Ozden, A., Golam Kibria, M., Seifitokaldani, A., Tan, C.S., Gabardo, C.M., Luo, M., et al. (2019). Catalyst synthesis under CO2 electroreduction favours faceting and promotes renewable fuels electrosynthesis. Nat. Catal.

6. Huang, J.E., Li, F., Ozden, A., Rasouli, A.S., Pelayo, F., Arquer, G. De, Liu, S., Zhang, S., Luo, M., Xu, Y., et al. (2021). CO 2 electrolysis to multi-carbon products in strong acid. Science *372*, 1074–1078.

7. Dinh, C.T., Burdyny, T., Kibria, G., Seifitokaldani, A., Gabardo, C.M., Pelayo García De Arquer, F., Kiani, A., Edwards, J.P., De Luna, P., Bushuyev, O.S., et al. (2018). CO2 electroreduction to ethylene via hydroxide-mediated copper catalysis at an abrupt interface. Science *360*, 783–787.

8. Vermaas, D.A., and Smith, W.A. (2016). Synergistic Electrochemical CO2 Reduction and Water Oxidation with a Bipolar Membrane. ACS Energy Lett. *1*, 1143–1148.

9. Kibria, M.G., Edwards, J.P., Gabardo, C.M., Dinh, C.T., Seifitokaldani, A., Sinton, D., and Sargent, E.H. (2019). Electrochemical CO2 Reduction into Chemical Feedstocks: From Mechanistic Electrocatalysis Models to System Design. Adv. Mater. *31*, 1–24.

10. Rabinowitz, J.A., and Kanan, M.W. (2020). The future of low-temperature carbon dioxide electrolysis depends on solving one basic problem. Nat. Commun. *11*, 10–12.

11. Ma, M., Clark, E.L., Chorkendorff, K.T.T.S.D., and Seger, B. (2020). Insights into the carbon balance for CO2 electroreduction on Cu using gas diffusion electrode reactor designs. Energy Environ. Sci. *13*, 977–985.

12. Ozden, A., Wang, Y., Li, F., Luo, M., Sisler, J., Thevenon, A., Rosas-Hernández, A., Burdyny, T., Lum, Y., Yadegari, H., et al. (2021). Cascade CO2 electroreduction enables efficient carbonate-free production of ethylene. Joule, 1–14.

13. Ma, M., Kim, S., Chorkendorff, I., and Seger, B. (2020). Role of ion-selective membranes in the carbon balance for CO2electroreduction: Via gas diffusion electrode reactor designs. Chem. Sci. *11*, 8854–8861.

14. Ma, S., Sadakiyo, M., Luo, R., Heima, M., Yamauchi, M., and Kenis, P.J.A. (2016). One-step electrosynthesis of ethylene and ethanol from CO 2 in an alkaline electrolyzer. J. Power Sources *301*, 219–228.

15. Verma, S., Hamasaki, Y., Kim, C., Huang, W., Lu, S., Jhong, H.R.M., Gewirth, A.A., Fujigaya, T., Nakashima, N., and Kenis, P.J.A. (2018). Insights into the Low Overpotential Electroreduction of CO2 to CO on a Supported Gold Catalyst in an Alkaline Flow Electrolyzer. ACS Energy Lett. *3*, 193–198.

16. Larrazábal, G.O., Strøm-Hansen, P., Heli, J.P., Zeiter, K., Therkildsen, K.T., Chorkendorff, I., and Seger, B. (2019). Analysis of Mass Flows and Membrane Cross-over in CO2 Reduction at High Current Densities in an MEA-Type Electrolyzer. ACS Appl. Mater. Interfaces *11*, 41281–41288.

17. Gabardo, C.M., O’Brien, C.P., Edwards, J.P., McCallum, C., Xu, Y., Dinh, C.T., Li, J., Sargent, E.H., and Sinton, D. (2019). Continuous Carbon Dioxide Electroreduction to Concentrated Multi-carbon Products Using a Membrane Electrode Assembly. Joule *3*, 2777–2791.

18. Jeng, E., and Jiao, F. (2020). Investigation of CO2 single-pass conversion in a flow electrolyzer. React. Chem. Eng. *5*, 1768–1775.

19. B. Endrodi, E. Kecsenovity, A. Samu, T. Halmagyi, S. Rojas-Carbonell, L. Wang, Y.Y. and C.J. (2020). High carbonate ion conductance of a robust PiperION membrane allows industrial current density and conversion in a zero-gap carbon. Energy Environ. Sci. *13*, 4098–4105.

20. Oener, S.Z., Foster, M.J., and Boettcher, S.W. (2020). Accelerating water dissociation in bipolar membranes and for electrocatalysis. Science *369*, 1099–1103.

21. Chen, Y., Vise, A., Klein, W.E., Cetinbas, F.C., Myers, D.J., Smith, W.A., Smith, W.A., Smith, W.A., Deutsch, T.G., and Neyerlin, K.C. (2020). A Robust, Scalable Platform for the Electrochemical Conversion of CO2to Formate: Identifying Pathways to Higher Energy Efficiencies. ACS Energy Lett. *5*, 1825–1833.

22. Yan, Z., Hitt, J.L., Zeng, Z., Hickner, M.A., and Mallouk, T.E. (2021). Improving the efficiency of CO2 electrolysis by using a bipolar membrane with a weak-acid cation exchange layer. Nat. Chem. *13*, 33–40.

23. Yang, K., Li, M., Subramanian, S., Blommaert, M.A., Smith, W.A., and Burdyny, T. (2021). Cation-Driven Increases of CO 2 Utilization in a Bipolar Membrane Electrode Assembly for CO 2 Electrolysis. ACS Energy Lett. *6*, 4291–4298.

24. Salvatore, D.A., Weekes, D.M., He, J., Dettelbach, K.E., Li, Y.C., Mallouk, T.E., and Berlinguette, C.P. (2018). Electrolysis of Gaseous CO2 to CO in a Flow Cell with a Bipolar Membrane. ACS Energy Lett. *3*, 149–154.

25. Delacourt, C., Ridgway, P.L., Kerr, J.B., and Newman, J. (2008). Design of an Electrochemical Cell Making Syngas (CO+H2) from CO2 and H2O Reduction at Room Temperature. J. Electrochem. Soc. *155*, B42.

26. Lee, G., Li, Y.C., Kim, J.Y., Peng, T., Nam, D.H., Sedighian Rasouli, A., Li, F., Luo, M., Ip, A.H., Joo, Y.C., et al. (2021). Electrochemical upgrade of CO2 from amine capture solution. Nat. Energy *6*, 46–53.

27. Singh, M.R., Kwon, Y., Lum, Y., Ager, J.W., and Bell, A.T. (2016). Hydrolysis of Electrolyte Cations Enhances the Electrochemical Reduction of CO2 over Ag and Cu. J. Am. Chem. Soc. *138*, 13006–13012.

28. Resasco, J., Chen, L.D., Clark, E., Tsai, C., Hahn, C., Jaramillo, T.F., Chan, K., and Bell, A.T. (2017). Promoter Effects of Alkali Metal Cations on the Electrochemical Reduction of Carbon Dioxide. J. Am. Chem. Soc. *139*, 11277–11287.

29. Sa, Y.J., Lee, C.W., Lee, S.Y., Na, J., Lee, U., and Hwang, Y.J. (2020). Catalyst-electrolyte interface chemistry for electrochemical CO2 reduction. Chem. Soc. Rev. *49*, 6632–6665.

30. Xu, Y., Edwards, J.P., Liu, S., Miao, R.K., Huang, J.E., Gabardo, C.M., O’Brien, C.P., Li, J., Sargent, E.H., and Sinton, D. (2021). Self-Cleaning CO 2 Reduction Systems: Unsteady Electrochemical Forcing Enables Stability . ACS Energy Lett. *6*, 809–815.

31. McGrogan, F.P., Swamy, T., Bishop, S.R., Eggleton, E., Porz, L., Chen, X., Chiang, Y.M., and Van Vliet, K.J. (2017). Compliant Yet Brittle Mechanical Behavior of Li2S–P2S5 Lithium-Ion-Conducting Solid Electrolyte. Adv. Energy Mater. *7*, 1–5.

32. Xie, K., Wu, H., Meng, Y., Lu, K., Wei, Z., and Zhang, Z. (2015). Poly(3,4-dinitrothiophene)/SWCNT composite as a low overpotential hydrogen evolution metal-free catalyst. J. Mater. Chem. A *3*, 78–82.

33. Xie, K., Qin, X., Wang, X., Wang, Y., Tao, H., Wu, Q., Yang, L., and Hu, Z. (2012). Carbon nanocages as supercapacitor electrode materials. Adv. Mater. *24*, 347–352.

34. Seo, S.J., Woo, J.J., Yun, S.H., Lee, H.J., Park, J.S., Xu, T., Yang, T.H., Lee, J., and Moon, S.H. (2010). Analyses of interfacial resistances in a membrane-electrode assembly for a proton exchange membrane fuel cell using symmetrical impedance spectroscopy. Phys. Chem. Chem. Phys. *12*, 15291–15300.

35. Rashid, N., Ahmad, M., and Ingole, P.P. (2020). Dendritic copper microstructured electrodeposits for e ffi cient and selective electrochemical reduction of carbon dioxide into C1 and C2 hydrocarbons. J. CO2 Util. *38*, 385–397.

36. Sisler, J., Shaihroz Kha, Ip, A.H., Jaffer, M.W.S.S.A., and Sargent, E.H. (2021). Ethylene Electrosynthesis : A Comparative Techno-economic Analysis of Alkaline vs Membrane Electrode Assembly vs CO2−CO− C2H4 Tandems. ACS Energy Lett. *6*, 997–1002.

37. Chen, Y., Lewis, N.S., and Xiang, C. (2020). Modeling the Performance of A Flow-Through Gas Diffusion Electrode for Electrochemical Reduction of CO or CO 2 . J. Electrochem. Soc. *167*, 114503.

38. Petr Vany ́sek (1996). ionic conductiVity and diffusion at infinite dilution. CRC Handb. Chem. Phys *96*, 5–98.

39. Aqion Table of Diffusion Coefficients. https://www.aqion.de/site/diffusion-coefficients.

40. Applin, K.R., and Lasaga, A.C. (1984). The determination of SO42-, NaSO4-, and MgSO40 tracer diffusion coefficients and their application to diagenetic flux calculations. Geochim. Cosmochim. Acta *48*, 2151–2162.

41. Sugisaki, M. (1975). Soret Coefficients and Heat of Transport of Polyvalent Electrolytes in an Aqueous Solution. Bull. Chem. Soc. Jpn., 2751.

42. Weisenberger, S., and Schumpe, A. (1996). Estimation of Gas Solubilities in Salt Solutions at Temperatures from 273 K to 363 K. AIChE J. *42*, 298–300.
